# Shaping the gradients driving phoretic micro-swimmers: influence of swimming speed, budget of carbonic acid and environment

**DOI:** 10.1140/epje/s10189-021-00026-9

**Published:** 2021-03-23

**Authors:** Nadir Möller, Benno Liebchen, Thomas Palberg

**Affiliations:** 1 Institute of Condensed Matter Physics, Johannes Gutenberg Universität, Staudinger Weg 7, 55128 Mainz, Germany; 2 Max Planck Graduade Center, Institute of Physics, Johannes Gutenberg Universität, Staudinger Weg 7, 55128 Mainz, Germany; 3 Institute for Condensed Matter Physics, Technische Universität Darmstadt, Hochschulstr. 8, 64289 Darmstadt, Germany

## Abstract

**Abstract:**

pH gradient-driven modular micro-swimmers are investigated as a model for a large variety of quasi-two-dimensional chemi-phoretic self-propelled entities. Using three-channel micro-photometry, we obtain a precise large field mapping of pH at a spatial resolution of a few microns and a pH resolution of $\sim 0.02~\hbox {pH}$ units for swimmers of different velocities propelling on two differently charged substrates. We model our results in terms of solutions of the three-dimensional advection–diffusion equation for a 1:1 electrolyte, i.e. carbonic acid, which is produced by ion exchange and consumed by equilibration with dissolved $\hbox {CO}_{2}$. We demonstrate the dependence of gradient shape and steepness on swimmer speed, diffusivity of chemicals, as well as the fuel budget. Moreover, we experimentally observe a subtle, but significant feedback of the swimmer’s immediate environment in terms of a substrate charge-mediated solvent convection. We discuss our findings in view of different recent results from other micro-fluidic or active matter investigations. We anticipate that they are relevant for quantitative modelling and targeted applications of diffusio-phoretic flows in general and artificial micro-swimmers in particular.

**Graphic Abstract:**

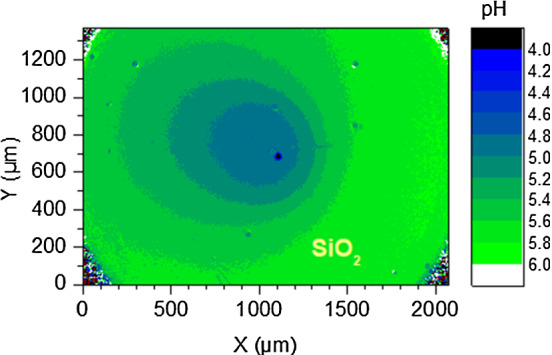

**Supplementary Information:**

The online version supplementary material available at 10.1140/epje/s10189-021-00026-9.

## Introduction

For small objects with no moving parts, propulsion by phoretic effects provides a fascinating route to swimming at low Reynolds number [1]. In the absence of body forces, phoretic motion relies on the slip velocity between the surface of an object and the adjacent fluid, which is often induced by a chemical potential gradient within the fluid directed across the surface [2]. Phoretic effects occur on the level of macroscopic surfaces, of small particles suspended in a fluid and even for molecular or ionic solutes. Prominent examples of phoretic effects include electrophoresis related to electric potential gradients [3, 4], thermo-phoresis based on temperature gradients [5–7] and diffusio-phoresis in concentration gradients [2, 8, 9]. In particular, the latter, which in general can feature neutral (chemi-phoretic) as well as charged (electrophoretic) contributions, can develop in a surprisingly wide variety of physical phenomena. Key applications of this important and abundant transport mechanism range from micro-fluidics to wastewater treatment [10–16]. Recently, it has attracted additional interest as an important underlying mechanism for chemo-taxis [17–19], non-equilibrium self-assembly [20–23] and artificial micro-swimming [24–32], where the gradients are self-generated locally and thus allow for self-organized, persistent directed motion [33]. If such swimmers propagate into a quiescent, pristine environment, they typically show a stationary directed propulsion superimposed by some rotational diffusion [34]. Their propulsion velocity depends on chemical reaction rates, but is further influenced by swimmer shape and size. Consequently, a slowdown of propulsion may result from fuel exhaustion or decomposition. More complicated modifications, like oscillatory propulsion, on–off switches, steering, cargo pickup and delivery, are conveniently realized employing oscillating chemical reactions [35, 36], as well as *via* additional local or global magnetic or optical fields [37–41].

In general, the targeted application of phoretic micro-swimmers and the modelling of their behaviour and swimming performance present considerable challenges. These are further increased in the case of collective behaviour like swarm formation and other dynamic phase transitions [32]. Several diffusio-phoretic self-propulsion mechanisms and the corresponding swimming performance have been characterized in great detail [24–31, 34–41]. However, experimental data on the shape and evolution of the relevant chemical concentration fields, as well as on their mutual phoretic and hydrodynamic couplings, are sparse. Therefore, up to now, most attempts for modelling had to rely on simplifying assumptions. A precise in situ characterization of the driving concentration gradients responsible for the propulsion (and interaction) of phoretic micro-swimmers as well as of their dependencies on boundary conditions is still highly desired. To advance our understanding beyond previous work, two conditions have to be met. First, a suitable gradient mapping technique has to accurately access the evolution of the concentration gradients of relevant chemicals and second the availability of a suitable, well-characterized experimental model representative for a large number of interesting systems. In the present paper, the first condition is met by employing a recently introduced photometric pH mapping technique and the second by studying pH-driven modular micro-swimmers.

Two different approaches to pH mapping have been reported in the literature. One is fluorescence intensity microscopy employing fluorophores as proxy for other chemicals [11, 42–44]. It features a good pH resolution and a very good (vertical) resolution [45, 59, 60], which can be further improved using two channel fluorescence and (confocal) microscopy [46, 47]. However, systematic artefacts due to chemical interference of other solutes or buffers with the fluorophore are quite common [48], and the need of strong fluorescence signals restricts the accessible pH range to typically one or two pH units. Micro-photometry on the other side provides height-averaged pH values. It exploits the colour contrast of standard universal pH indicators providing excellent chemical stability and access to a much broader pH range (of up to ten orders of magnitude in proton concentration). In multi-channel photometry [57] one calibrates the monotonously changing intensity ratios of each two RGB channels of a standard consumer DSLR to pH. In such ratios, no small numbers occur, providing a significantly improved accuracy for the calibration over against fluorescence or single-channel photometry [49]. Niu et al. [57] reported a resolution of $<0.1$ pH units/8 $\upmu$m in their pump gradient measurements. We here employ their approach for mapping height-averaged pHs with a further improved pH resolution of $\sim$ 0.02 pH units per $6\,\upmu \hbox {m}$.

**Fig. 1:**
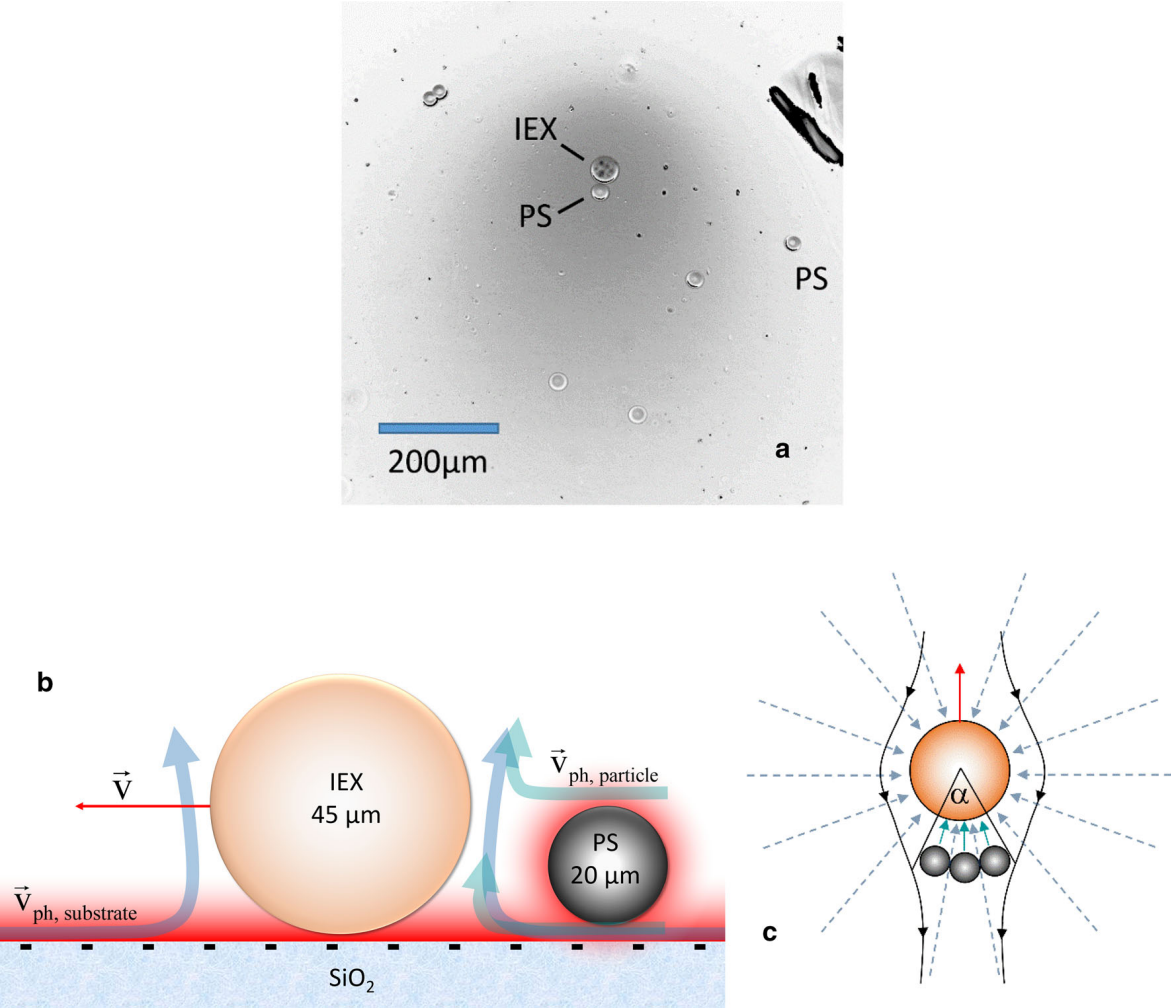
Modular phoretic micro-swimmers driven by a self-generated pH field: **a** single-channel photometric image of a modular micro-swimmer comprising one ion exchange resin (IEX) and one cargo colloid (PS). The ion exchange resin bead exchanges residual cations for protons. The resulting excess concentration of carbonic acid disperses in the surroundings creating a monotonously decaying concentration field, i.e. large-scale pH gradients. Dark image parts denote lower pH. **b** To scale side view sketch of the assembly and propulsion mechanism. Note the large extension of the electric double layers under realistic salt-free conditions (red shaded regions). The gradients induce a symmetric phoretic solvent flow ($\mathbf{v} _{\mathrm{ph, substrate}}$, light blue) along the charged $\hbox {SiO}_{{2}}$ substrate. Flows converge at the mobile IEX. They collect and trap charged polystyrene particles or anionic IEX beads as cargo. Across the cargo surface, the local gradient induces additional phoretic solvent flows ($\mathbf{v} _{\mathrm{ph, particle}}$, green). These break the flow symmetry and drive propulsion of the complex at a velocity **v** (red). **c** Top view sketch of different flows. Colours as in **b**. The swimming velocity increases stepwise with the number of cargo particles coupled to the IEX. The black, arrowed lines denote streamlines of the solvent relative to the swimmer. Interference with the solvent defines a maximum angle $\alpha$ to host some cargo resulting in a cargo size-dependent maximum cargo number and, hence, velocity

Regarding the second condition, micro-swimmers come in two main varieties. For single-component bulk swimmers (like catalytic Janus swimmers), the relevant chemicals are typically produced and/or consumed at or close to the particle surface. Their self-propulsion is dominated by the (co-moving) concentration gradients near the swimmer surface. The tail of these fields at large distances, however, is of crucial importance, e.g. for cargo collection, mutual swimmer interactions and chemo-taxis [37, 50, 51]. A second group of phoretic swimmers, so-called modular swimmers, uses these large-scale gradients and the associated flow fields to self-assemble and, then, exploits local gradients and flows to propel [52]. In particular, pH gradient-driven modular systems appear to be a suitable model for a large variety of chemi-phoretic swimmers [25, 30, 53, 54]. Figure 1a shows the pH field (grey shaded) around such a swimmer propelling into an aqueous solvent. The swimmer is composed of an ion exchange bead and a cargo particle hydrodynamically coupled to its back. The assembly and propulsion mechanism is schematically shown in Fig. 1b, c. As can be seen in Fig. 1a, these gravitationally settled systems readily submit to simultaneous studies on different scales. On the one side, we observe the assembly of swimmers occurring in large-scale pH gradients and the co-motion of these gradients, as the swimmers propel over large distances. Moreover, they can mediate swimmer–swimmer interactions *via *chemo-taxis [55, 56]. On the other side, we can determine local pH gradients along the cargo contour. These initiate additional phoretic flows along the cargo surface, thus breaking the overall flow symmetry and pushing the assembled complex forward with speed and direction set by cargo number and arrangement.

In the present paper, we employ this established model system of modular swimmers based on mobile ion exchange resin (IEX) beads settled onto a negatively charged substrate and exchanging residual cations for protons. Similar as in the case of fixed IEX functioning as pumps [57–61], realistic salt-free conditions [66] provide steep gradients. Exchangeable cations either originate from the substrate or derive from the $\hbox {CO}_{2}$-saturated water and ion exchange generates concentration fields of carbonic acid, $\hbox {HCO}_{{3}}^{{-}}\hbox {H}^{{+}}$. The involved mechanisms for assembly and propulsion are in general well understood, and the swimming performance of the resulting self-propelling aggregates has been explored in great experimental detail as a function of cargo number, size and charge as well as the possibilities of steering by manipulating the cargo arrangement [25, 30, 52–54]. Swimming speed in general was found to be proportional to the cargo number and the cargo electrophoretic mobility as well as to the pH gradient calculated for the assembly distance. An interesting open question, however, arose from the observation that swimmers of identical components and same cargo number and assembled at the same distance showed different speeds on different substrates [53]. Such an influence of the substrate type is not expected from models for the chemical fields based purely on diffusion. It was, however, readily reproduced in the present experiments. One therefore suspects the presence of several feedback mechanisms impacting on the shape of the relevant chemical fields:

First, the centre of mass motion of the source within the solvent breaks the isotropy of the concentration fields. For a point source propelling at constant velocity into a quiescent, pristine solvent, one expects a concentration decay, which is faster at the front than at the back. A corresponding “trail formation” behaviour is also known from environmental science for the equivalent case of a stationary pollution source in a homogeneously moving solvent or atmosphere [62]. Second, a mutual coupling may exist between the phoretic flow along the substrate and the solute diffusion. Most work on swimming so far neglected this effect, due to the scarceness of experimental evidence and formidable challenges to modelling (for a recent exception, see [63]). Advection-modified dispersion of chemicals is difficult to detect, and most studies, in fact, assume undistorted and stationary gradients solely governed by reaction rates and simple diffusion [26–28]. However, they have a considerable influence in the case of micro-fluidic pumps [60, 61]. A third complication arises when stable products of the ion exchange reaction accumulate in the cell. This leads to a slow change in background concentrations and may weaken the gradients An important example would be cation exchange in the presence of NaCl. On the other side, additional chemical reactions lead to a drain or loss term in the diffusion equations and can result in a steepening of gradients. These are provided for water-based swimmers by the buffering function of the $\hbox {CO}_{2}/\hbox {H}_{{2}}\hbox {CO}_{{3}}/\hbox {HCO}_{{3}}^{{-}}$ system [64, 65], which is retained even under nominally salt-free conditions [4, 66]. Finally, a possible diffusio-phoretic slowing of solute dispersion may occur for multi-ion systems [8, 13]. Since all these modifications altering the gradient magnitude at the surface generating the phoretic propulsion are mutually coupled, they close the feedback loop and change the resulting propulsion speed.

In what follows, we provide a comprehensive characterization of height-averaged pH gradients forming around modular micro-swimmers using multi-channel micro-photometry. We introduce a model based on the solution of the three-dimensional advection–diffusion equation for a moving point source. This is equivalent to a stationary source in a solvent, which moves with a constant speed. The model accounts for swimmer speed, chemical reaction rates, chemical diffusivity and constant background electrolyte concentration set by $\hbox {CO}_{2}$ buffering on the evolution of the pH field. It is further adapted to the experimental boundary conditions by considering the finite cell height and the height averaging effects of photometry. Electro-osmotically driven solvent flow fields, however, are not yet explicitly considered. We then compare our results to our model and derive estimates of reaction rates and an effective diffusivity, which is independent of swimmer speed but is decreased as compared to expectation for the more highly charged substrate. From this and complementary experiments on stationary IEX pumps, we finally can discuss the most significant gradient shaping processes and feedback mechanisms present in our systems. In fact, a major finding of this work will be an unequivocal establishment of a subtle but significant feedback of the electro-osmotically driven solvent flows on the diffusion of the chemicals.

## Modelling

### Profiles for stationary sources

To model the spatial and temporal evolution of the pH fields, let us consider the following diffusion equation of an effective chemical concentration field $c(\mathbf{r} , t)$ in stationary solution which is produced by a point source a position $\mathbf{r} _{{0}}$, emitting the chemical with a rate, $\upmu ^{{+}}$(t), which may generally depend on time [55]:

1 $$\begin{aligned} \frac{\partial c({\mathbf{r}},t)}{\partial t}=D\Delta c({\mathbf{r}},t)-\mu ^{-}c({\mathbf{r}},t)+\mu ^{+}(t)\delta ({\mathbf{r}}-{\mathbf{r}}_{0} ) \end{aligned}$$

Here, *D* is the relevant effective diffusion coefficient for the chemical. We have also introduced a sink term which is proportional to the rate $\upmu ^{{-}}$ and which may account for bulk reactions. We here consider a 1:1 electrolyte (carbonic acid), for which Nernst’s mutual diffusion coefficient applies:

2 $$\begin{aligned} D=\frac{2D_{+} D_{-} }{D_{+} +D_{-} }, \end{aligned}$$

where the subscripts $+$ and - denote the free diffusion coefficients of the cation and the anion, respectively. For example, for carbonic acid, we have at standard conditions and infinite dilution $D_{H+} = 9310\,\upmu \hbox {m}^{{2}}~\hbox {s}^{{-1}}$ and $D_\mathrm{HCO3-}= 1180\,\upmu \hbox {m}^{{2}}~\hbox {s}^{{-1}}$ [67], which yields $D = 2096\upmu \hbox {m}^{{2}}~\hbox {s}^{{-1}}$ for Nernst’s mutual diffusion coefficient.

For pump experiments, $\mathbf{r} _{{0}}$ is fixed, and the production rate is assumed constant and to start instantaneously at $t = 0$, i.e. $\upmu ^{{+}}(t) = \upmu ^{{+}} \Theta (t)$, where $\Theta (t)$ denotes the unit step function. The diffusion equation (1) can be considered in $d = 1, 2, 3$ spatial dimensions, and its formal solution for *d* dimensions in infinite space is given by [55]:

3 $$\begin{aligned} c({\mathbf{r}},t)= & {} \mu ^{+}\int _0^t \hbox {d}t' \,\,\frac{1}{(4\pi D\left| {t-t' } \right| )^{d/2}}\nonumber \\&\exp \left( {-\,\frac{({\mathbf{r}}-{\mathbf{r}}_{0} )^{2}}{4D\left| {t-t' } \right| }-\mu ^{-}\left| {t-t'} \right| } \right) +c_{\infty }. \end{aligned}$$

Here, $c_{\inf }$ denotes the background concentration. In experimental situations cylindrical cells of radius *R* and height *H* with $\hbox {R}>> \hbox {H} > a$ and settled sources at $\mathbf{r} _{{0 }}= (0,0,a)$ are often approximated as effectively two-dimensional [14, 16, 61] (where in our experiments, $a = R_{IEX}$, the IEX radius). We note that a stationary solution for two dimensions is only obtained for very large *R* and a non-vanishing loss rate, $\upmu ^{{-}} > 0$. If, however, $\upmu ^{{-}} = 0$, (e.g. exchanging Na$^{+}$Cl$^{-}$ to HCl) or R is finite the chemical accumulates, i.e. $c_{{\infty }}= c_{{\infty }}(\hbox {t})$, and no stationary solution can be reached [55]. For the present experiments, we consider three dimensions. In the simple case of a constant production rate, zero background concentration and zero loss rate, Eq. (3) can be written as:

4 $$\begin{aligned} c({\mathbf{r}},t)=\frac{\mu ^{+}}{4\pi D\left| {{\mathbf{r}}-{\mathbf{r}}_{0} } \right| }\text{ erfc }\left( {\frac{\left| {{\mathbf{r}}-{\mathbf{r}}_{0} } \right| }{2\sqrt{Dt} }} \right) , \end{aligned}$$

where erfc$(x) = 1-erf(x)$ is the complementary error function. In the case of a finite loss rate, $\upmu ^{{-}}$, and a finite background concentration, $c_{{\infty }}> 0$, the resulting equation for the chemical field is more complicated. However, if $\upmu ^{{-}}t$ “ 1, which is typically fulfilled in our experiments over the relevant observation timescales, we obtain the following expression for the 3D concentration field:

5 $$\begin{aligned} c({\mathbf{r}},t)\approx c_{\infty } +\frac{\mu ^{+}}{4\pi D}\,\upvarphi ({\mathbf{r}},t), \end{aligned}$$

where the first term on the right-hand side is the background concentration and the second term is the excess concentration of the chemical, where

6 $$\begin{aligned} \upvarphi ({\mathbf{r}},t)=\frac{1}{\left| {{\mathbf{r}}-{\mathbf{r}}_{0} } \right| }\,\text{ erfc }\left( {\frac{\left| {{\mathbf{r}}-{\mathbf{r}}_{0} } \right| }{2\sqrt{Dt} }} \right) \exp (-\mu ^{-}t). \end{aligned}$$

Here, the excess concentration depends linearly on production rate, which is consistent with previous micro-swimmer studies [26, 27, 38, 59], and the exponential describes the impact of loses by bulk reactions or precipitation. Note that both a small diffusivity and a finite loss rate will steepen the resulting concentration profile. At large distances the concentration approaches the bulk value $c_{{\infty }}$. In the present experiments, the relevant chemical is carbonic acid. For the aqueous pH field in free 3D space, it follows from Eqs. (5) and (6) that:

7 $$\begin{aligned}&\text{ pH }({\mathbf{r}},t)\approx -\log _{10}\nonumber \\&\quad \left( {c_{\infty } +\frac{\mu ^{+}}{4\pi D\left| {{\mathbf{r}}-{\mathbf{r}}_{0} } \right| }\,\text{ erfc }\left( {\frac{\left| {{\mathbf{r}}-{\mathbf{r}}_{0} } \right| }{2\sqrt{Dt} }} \right) \exp (-\mu ^{-}t)} \right) .\nonumber \\ \end{aligned}$$

We took care to ensure a homogeneous pristine environment of realistic salt-free water, buffered by dissolved $\hbox {CO}_{2}$ to $\hbox {pH}_{{\infty }} \approx 5.5$. In this ideal case, $\upmu ^{{-}}$ is finite and regulated by the dissociation equilibrium, and $c_{{\infty }}$ is a constant. However, for other pumping experiments conducted in the presence of additional neutral salts like *NaCl*, one expects a slow acidification of the water and an additional interference with the carbonate equilibrium [see below, Eqs. (16)–(18)]. Even then, for sufficiently large cells, the pH profile can remain quasi-stationary, such that $c_{{\infty }}= c_{{\infty }}$(t) remains valid for Eq. (7).

### Profiles for moving sources

Let us now discuss how the chemical concentration field changes when the source, i.e. the micro-swimmer, which produces it, moves in unbound 3D space at a constant velocity **v**. We choose a coordinate system such that $\mathbf{e} _{\mathrm{x}}\vert \vert \mathbf{v}$. The diffusion equation for constant emission rate then reads:

8 $$\begin{aligned} \frac{\partial c({\mathbf{r}},t)}{\partial t}=D\Delta c({\mathbf{r}},t)-\mu ^{-}c({\mathbf{r}},t)+\mu ^{+}\delta ({\mathbf{r}}-{\mathbf{r}}_{0} -{\mathbf{v}}t).\nonumber \\ \end{aligned}$$

We now obtain a steady state solution, which reads [55]:

9 $$\begin{aligned} c({\mathbf{r}})=c_{\infty } +\frac{\mu ^{+}}{4\pi D}\upvarphi ({\mathbf{r}}), \end{aligned}$$

where the shape of the excess concentration field now is independent of time

10 $$\begin{aligned} \upvarphi ({\mathbf{r}})=\frac{1}{\left| {{\mathbf{r}}-{\mathbf{r}}_{0} } \right| }\exp \left( {-\,\kappa \left| {{\mathbf{r}}-{\mathbf{r}}_{0} } \right| -\frac{{\mathbf{v}}\cdot ({\mathbf{r}}-{\mathbf{r}}_{0} )}{2D}} \right) ,\nonumber \\ \end{aligned}$$

Here, the effective screening constant, $\kappa$, depends on the swimming speed:

11 $$\begin{aligned} \kappa =\sqrt{\frac{\mu ^{-}}{D}+\frac{{\mathbf{v}}^{2}}{4D^{2}}}. \end{aligned}$$

From Eq. (9) we expect a steep outward decrease in concentration close to the source and some saturation effects far away. For small loss rates, the velocity dependence of the exponential yields a steeper slope at the front than at the back.

### Geometric effects

There are two geometric effects present in experiments, regardless whether the source is stationary or moving: geometric confinement and height averaging.

**Fig. 2:**
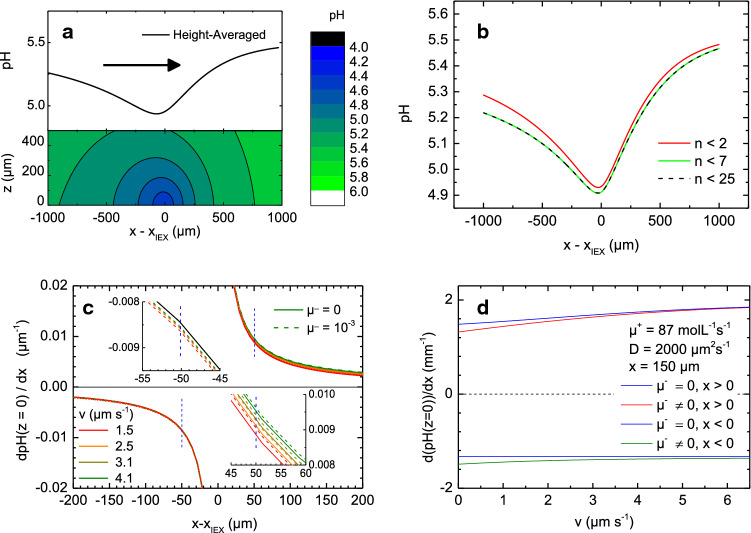
Theoretically expected pH field behaviour. **a** Bottom: pH distribution in the *x*-*z* plane calculated using Eqs. (10) and (12) for $\upmu ^{{+}}= 87~\hbox {mol L}^{{-1}}~\hbox {s}^{{-1}}$, $\upmu ^{{-}} = 10^{{-3 }}~\hbox {s}^{{-1}}$, $c_{{\infty }} = 3.16~\hbox {mol L}^{{-1}}$ corresponding to $\hbox {pH} = 5.5$, and $D = 2000~\upmu \hbox {m}^{{2}}~\hbox {s}^{{-1}}$, $\hbox {v} = 4.1~\upmu \hbox {m}~\hbox {s}^{{-1}}$, $\hbox {H} = 500~\upmu \hbox {m}$ and $n = 25$. Top: height-averaged pH profile along the swimming direction $\mathbf{v} \vert \vert \mathbf{e} _{\mathrm{x}}$. The arrow indicates the propulsion direction. **b** Convergence of the averaging procedure. Height-averaged pH profiles for different *n*as indicated **c** Ground slopes calculated from Eq. (15) for $\upmu ^{{-}} = 0$ (solid lines) and $\upmu ^{{-}} = 10^{{-3 }}~\hbox {s}^{{-1}}$ (dashed lines), as well as for different velocities colour coded as defined in the key. The insets magnify regions about $\vert x-x_{\mathrm{IEX}}\vert = 50~\upmu \hbox {m}$, corresponding to typical cargo distances. **d** Characteristic velocity dependence of the ground slopes at a fixed distances of $\vert x- x_{\mathrm{IEX}}\vert = 150~\upmu \hbox {m}$. Shown are the front and the back slopes for zero as well as for finite loss rate ($\upmu ^{{-}} = 10^{{-3 }}~\hbox {s}^{{-1}}$) with different combinations colour coded as defined in the key

(i) First, while in all our experiments the lateral cell extension is sufficiently large to neglect lateral confinement, this does not hold for the vertical cell extension. We implement the effects of the vertical confinement by introducing a series of mirror sources. The proton concentration field within the domain $0< \hbox {z} < \hbox {H}$ is then expressed as:
  12 $$\begin{aligned} c({\mathbf{r}},t)= & {} c_{\infty } +\frac{\mu ^{+}}{4\pi D}\sum \limits _{n=0}^\infty {\upvarphi \left( {{\mathbf{r}}+\left( {2nH+R_{IEX} } \right) {\mathbf{e}}_{z} ,t} \right) } \nonumber \\&+\upvarphi \left( {{\mathbf{r}}+\left( {2nH-R_{IEX} } \right) {\mathbf{e}}_{z} ,t} \right) , \end{aligned}$$
  where depending on the experiment considered either Eq. (6) or Eq. (10) has to be used for $\phi (\mathbf{r} )$. The limit of the series is analytically difficult to solve; however, contributions for large *n* rapidly decay to 0, so that the sum is numerically evaluated up to $n = 6$, after which $c({\mathbf{r}},t)$ does not significantly change (see Fig. 2b).
(ii) Second, the photometric experiments are performed in transmission geometry. Thus, for each lateral position (x,y), the measurable pH field is an average along ${\mathbf{e}}_{z}$.
  13 $$\begin{aligned} \text{ pH}_{\text{ z,Av }} (x,y)=\frac{1}{H}\int _0^H {\text{ pH }(x,y,z)\text{ d }z} . \end{aligned}$$

To illustrate the effects of confinement and of averaging, we display an exemplary stationary x-z distribution of the pH for a cell with $\hbox {H}=500~\upmu \hbox {m}$ in the lower part of Fig. 2a. This distribution was calculated with $\mathbf{e} _{\mathrm{x}}\vert \vert \mathbf{v}$ for a set of typical parameters ($\upmu ^{{+}}= 87~\hbox {mol L}^{{-1}}~\hbox {s}^{{-1}}$, $\upmu ^{{-}} = 10^{{-3}}~\hbox {s}^{{-1}}$, $\hbox {v} = 4.1~\upmu \hbox {m s}^{{-1}}$, $c_{{\infty }} = 3.16~\hbox {mol L}^{{-1}}$ corresponding to $\hbox {pH} = 5.5$, and $D = 2000~\upmu \hbox {m}^{{2}}~\hbox {s}^{{-1}}$) in Eqs. (10) and (12) with $n = 25$. The upper part of Fig. 2a shows the height-averaged profile. In Fig. 2b we show the fast convergence of the calculations with increasing *n*. Here, we perform the average outside of the IEX, i.e. for $\left| {{\mathbf{r}}-{\mathbf{r}}_{{\mathbf{0}}} } \right| <25\,\upmu \hbox {m}$.

### Slopes

The local gradient of a pH field (the slope) at the location of a charged surface determines the magnitude of the slip velocity between the solvent and this surface. In the general case, the slope measurable in our experiments follows from the (numerical) derivative of the profiles calculated above. We here approximated it analytically using:

14 $$\begin{aligned} \frac{\partial \text{ pH}_{\exp } }{\partial x}=\frac{c-c_{\infty } }{\ln (10)c}\left( {\frac{(x-x_{0} )}{\left| {{\mathbf{r}}-{\mathbf{r}}_{0} } \right| ^{2}}+\kappa \frac{(x-x_{0} )}{\left| {{\mathbf{r}}-{\mathbf{r}}_{0} } \right| }+\frac{\text{ v }}{2D}} \right) \end{aligned}$$

For estimating slip velocities at the surface of sedimented cargo particles (c.f. Eqs. (S1) and (S2), in the ESM), the special case of $\mathbf{r} = (x,0,0)$ and $\mathbf{r} _{{0}} = (0,0,0)$ is relevant. From this we find for the ground slope:

15 $$\begin{aligned} \frac{\partial \text{ pH }}{\partial x}=\frac{c-c_{\infty } }{\ln (10)c}\left( {\frac{1}{x}\,\,\,+\,\,\,\frac{x}{\left| x \right| }\sqrt{\frac{\mu ^{-}}{D}+\frac{\text{ v}^{2}}{4D^{2}}} \,\,\,+\,\,\,\frac{\text{ v }}{2D}} \right) ,\nonumber \\ \end{aligned}$$

Note the sign change at the source position. Interestingly, both Eqs. (14) and (15) predict qualitative differences in the dependence on velocity for the front and the back slopes. In Fig. 2c, we display the ground slopes as a function of distance ($x-x_{\mathrm{IEX}})$ as calculated from Eq. (15) for $D = 2000~\upmu \hbox {m}^{{2}}~\hbox {s}^{{-1}}$, $\upmu ^{{+}} = 87~\hbox {mol L}^{{-1}}~\hbox {s}^{{-1}}$ and $c_{{\infty }} = 3.16~\hbox {mol L}^{{-1}}$. For various swimming speeds, we compare results for $\upmu ^{{-}} = 0$ (solid lines) to results for $\upmu ^{{-}} = 10^{{-3 }}~\hbox {s}^{{-1}}$ (dashed lines). Both front and back slopes are small at large distances from the IEX, $\vert x-x_{\mathrm{IEX}}\vert$. With decreasing distance to the source, the slopes gain increasingly steepen and finally diverge. Further, irrespective of the swimming speed, all back slopes approximately coincide, while this is not the case for the front slopes. The insets of Fig. 1c magnify this behaviour for a region about $\vert x$-$x_{\mathrm{IEX}}\vert = 50~\upmu \hbox {m}$, corresponding to a typical cargo distance. The back slopes coincide exactly at zero loss rate, at finite loss rate a minute velocity dependence is visible. The front slopes, however, increase both with increasing velocity and with increasing $\upmu ^{{-}}$.

The velocity dependence of the ground slope at a fixed positions of $\vert x$-$x_{\mathrm{IEX}}\vert = 150~\upmu \hbox {m}$ is displayed in Fig. 2d. At zero loss rate the back slope (blue) is independent of velocity, as the terms within the square root of Eq. (15) cancel with the last term. At the same time, the front slope (red) initially increases linearly with velocity. The increase, however, slows as the onset of saturation effects approaches the chosen *x* for larger velocities. This finally yields a maximum at very large velocities (not shown). Further, in the limit $\hbox {v} \rightarrow 0$, both front and back slope take the same absolute value. Conversely, for finite loss rates, both back slope (olive) and front slope (violet) show larger absolute values at low velocities and asymptotically approach the $\upmu ^{{-}} = 0$ behaviour at large velocities. This testifies that saturation effects now become visible at the chosen *x*. Remarkably, the slopes of height-averaged pH profiles show a qualitatively identical behaviour at only slightly lower values. The distance of maximum front slope, however, is located closer to the IEX. Readers interested in further explorations on the predictions and possible consequences of Eqs. (14) and (15) are referred to the ESM, Sect. 2.

## Materials and methods

### Sample preparation

We assemble our swimmers from ion exchange resin beads and charged colloidal spheres as cargo, both suspended in different aqueous electrolytes, and settled to the charged substrates. The observation cell was constructed from circular poly-methyl-methacrylate (PMMA; Perspex®) rings with diameter of $R = 12.5~\hbox {mm}$ inner radius attached to a microscopy slide by hydrolytically inert epoxy glue (UHU plus sofortfest, UHU GmbH, Germany) and dried for 24 h before use. Standard ring height was $H \approx 0.5~\hbox {mm}$, slightly varying with glue layer thickness. Commercial soda lime glass slides of hydrolytic class 3 (VWR International, Germany) served as substrates. These were washed with 1% alkaline solution (Hellmanex®III, Hellma Analytics) under sonication for 30 min, then rinsed with tap water and subsequently washed with doubly distilled water for several times. Substrate surface potentials were determined using Doppler velocimetry under realistic salt-free conditions [58]. Their zeta potential was $\zeta = -\,138.2 \pm 8.0~\hbox {mV}$, corresponding to an electro-osmotic mobility of $\upmu _{\mathrm{eo}} = 11 \pm 0.7~\upmu \hbox {m}/\hbox {s}/ \hbox {V}/\hbox {cm}$. In addition, cells built using Perspex®slides ($\zeta = -\,58.7 \pm 4.4~\hbox {mV}$, $\upmu _{\mathrm{eo}} = 4.8 \pm 0.4~\upmu \hbox {m}/\hbox {s} / \hbox {V}/\hbox {cm}$), cut to $75 \times 25~\hbox {mm}^{{2}}$ from larger sheets were used for the substrate-dependent measurements. These were prepared using the same procedure as the soda lime glass slides.

The IEX beads are micro-gel-based cationic ion exchange resin sphere of diameters $2a = 45\pm 1 \mu \hbox {m}$ measured by density matching ($\hbox {CGC}50\times 8$, Purolite Ltd, UK). For swimming, we loosely distributed a few IEX spheres inside the cell. To study solvent flow at stationary pumps, a single bead was glued mid-cell on the bottom substrate. The cell was then covered by a second microscopy side (either glass or Perspex®) bearing two small holes, to avoid contamination by dust and simultaneously allow maintaining equilibration with ambient $\hbox {CO}_{2}$. Through these, we subsequently fill the cell with colloidal particle suspension without remaining bubbles.

The cargo particle suspensions contained sulphate-stabilized PS spheres of diameter $15.2 \mu ~\hbox {m}$ or $20.1~\upmu \hbox {m}$ (from TEM) and an electrophoretic mobility of $\upmu _{\mathrm{ep}} = 2.1~\upmu \hbox {m}/\hbox {s} / \hbox {V}/\hbox {cm}$ (PS/Q-F-L1488 and PS/Q-F-L2619, MicroParticles GmbH, Germany). The commercial stock PS particle suspensions were first diluted, then kept on ion exchange resin (Amberlite K306, Carl Roth GmbH $+$ Co. KG, Karlsruhe, Germany) for desalination. Prior to use they were diluted further with doubly distilled water and a small amount of a diluted mixture of two standard pH indicator fluids was added (pH 4.0–10.0 Universal Indicator, MERCK; pH 0–5.0 pH indicator solution, Sigma-Aldrich). Indicator concentrations of 200–$500~\upmu \hbox {mol L}^{{-1}}$ provide good colour contrast over a large pH range but avoid interference with swimming performance. We then left the suspensions to equilibrate in contact with ambient air to obtain so-called realistic salt-free conditions [66].

### Ion exchange

Gradients for phoretic propulsion are generated by ion exchange. We explicitly note that realistic salt-free conditions do not imply absence of any ions. Rather, there still are the counter-ions of any added particle or surface, (here assumed to be $\hbox {H}^{{+}}$), and further ions from different sources. These include residual stray ions (e.g. $\hbox {Na}^{{+}}$ or $\hbox {K}^{{+}}$), $\hbox {H}^{{+}}$ and $\hbox {OH}^{{-}}$ (from water hydrolysis), as well as $\hbox {H}^{{+}}$, bicarbonate anions $\hbox {HCO}_{{3}}^{{-}}$, and neutral carbonic acid molecules $\hbox {H}_{{2}}\hbox {CO}_{{3}}$, from the atmospheric $\hbox {CO}_{2}$ contamination [4, 58, 64, 65, 68]

In our experiments, the ions to be exchanged are $\hbox {Na}^{{+}}$ and $\hbox {K}^{{+}}$ either from the glass substrate or left over from the washing procedures. These dissolve under reaction with $\hbox {CO}_{2}$ to form alkali carbonates. For cleaning protocols and boundary conditions close to the ones employed her, Niu et al. had estimated their concentration to be $10^{{-8}}$–$10^{{-7}}~\hbox {mol L}^{{-1}}$ [57]. This is low enough to exclude any influence on the carbonate dissociation equilibrium [64, 65]. At the resin beads, salt cations, $B^{+}$, are exchanged for protons forming an acid with the residual Anion, $A^{{-}}$. We assume this process to be reaction-controlled and not limited by transport of the salt towards the IEX:

16 $$\begin{aligned} R-H+A^{-}B^{+}\Rightarrow R-B+A^{-}H^{+}, \end{aligned}$$

where *R* denotes the polymeric resin backbone. It results in a surplus of acid close to the IEX, dispersing *via* diffusion. For bicarbonate:

17 $$\begin{aligned} \hbox {R}-\hbox {H}\,+\,\hbox {Na}^{+}+\hbox {HCO}_{3}^{-}\Rightarrow \hbox {R}-\hbox {Na}\,+\,\hbox {H}^{+}+\hbox {HCO}_{3} ^{-},\nonumber \\ \end{aligned}$$

This produces a surplus of carbonic acid close to the resin, which is part of the dissociation scheme of $\hbox {CO}_{2}$. The carbonate-related chemical reactions in the realistic salt-free aqueous solution are:

18 $$\begin{aligned}&\hbox {H}_{2}\hbox {O}\,\,\underset{K_{-1} }{\overset{K_{1} }{\rightleftarrows }}\,\,\hbox {H}^{+}+\,\,\,\hbox {OH}^{-} \nonumber \\&\hbox {H}_{2} \hbox {CO}_{3} \,\,\underset{K_{-2} }{\overset{K_{2} }{\rightleftarrows }}\,\,\hbox {H}^{+}+\,\,\,\hbox {HCO}_{3}^{-} \nonumber \\&\hbox {H}_{2} \hbox {CO}_{3} \,\,\underset{K_{-3} }{\overset{K_{3} }{\rightleftarrows }}\,\,\hbox {CO}_{2} +\,\,\,\hbox {H}_{2} \hbox {O}. \end{aligned}$$

$K_{\mathrm{i}}$ and $K_{-i}$ ($i=1, 2, 3$) are the corresponding forward ($\hbox {s}^{{-1}}$) and backward ($\hbox {m}^{{3 }}~\hbox {s}^{{-1}})$ kinetic constants. According to the solubility and partial pressure of $\hbox {CO}_{2}$ in standard air at $25\,^{\circ }\hbox {C}$, the concentration of $\hbox {CO}_{2}$ in realistic salt-free water is $1.18\times 10^{{-5}}~\hbox {mol L}^{{-1}}$ [64] which amounts to $\hbox {pH} \approx 5.5$. Note, however, that this equilibrium may shift in dependence on temperature and pressure as well as for simultaneously present further salts or acids [64, 65]. The surplus of carbonic acid, therefore, can distribute diffusively and can decay through successive reaction.

### Swimmer tracking

After filling the cell with IEX beads and cargo particle suspension, the modular swimmers self-assemble, start propelling, pick up cargo one by one and further accelerate [53]. We monitor swimmer propulsion by video tracking and the concentration of carbonic acid *via* high-resolution pH mapping. We mount the cell to the x-y stage of an inverted microscope (DMIRBE, Leica, Germany) allowing for uniform Köhler illumination ($\Delta I(x,y) / <I>\, \le 0.015$) and observation in transmission with two cameras simultaneously. A standard video camera at 60fps records tracking videos at $5\times \hbox { or } 10\times$ magnifications using and analysed using custom-written tracking algorithms [14]. Randomness in the self-organized swimmer formation presents a major challenge to the observation of isolated and undisturbed swimmer trajectories. On the one side, the initial propulsion direction may be such that it crosses other swimmer paths; on the other side, cargo pick-up alters direction and velocity and frequent cargo rearrangements introduce small irregularities in the propulsion, which are readily observable in the pH field shape and often render field fitting inconclusive. In particular, for fast swimmers, the transition from excess acid concentrations to the smooth low background concentration is frequently disturbed. For a selection of velocity measurements and more details on swimmer selection criteria for pH mapping, see chapter 3 of the ESM.

### pH mapping

For pH mapping, we employ a further refined version of the general photometric approach reported by Liu et al. [57]. We observed the sample with a consumer DSLR (D700, Nikon, Japan) using a 0.63x mounting tube featuring a FX-format CMOS sensor ($36.0 \times 23.9~\hbox {mm}^{{2}}$) and allowing for complete turn-off of any white balance algorithms. The $4.256 \times 2.832 = 12.87$ Mpix on the sensor are arranged in a Bayer pattern for the three channels resulting in four pixel dots, defining the maximum spatial resolution. We convert the initially captured 14-bit RAW format to TIFF for further processing. A custom-written python script achieves pixel-wise splitting of the intensity readings of each colour channel (RGB) [57]. For each channel, we bin the data in blocks containing 6x6 pixels of the same channel. Then, we calculate the block mean absorbance, *A*, for this channel, using Lambert Beer’s law:

19 $$\begin{aligned} A=-\ln \left( {\frac{I(\mu ,c,d)}{I_{0} }} \right) =\mu \,\,c\,\,d \end{aligned}$$

Here, *I* and $I_{0}$ denote the light intensity transmitted through either the sample or a water filled reference cell, respectively, and $\upmu$ is the wave length-dependent absorption coefficient. Indicator concentration, *c*, and cell thickness, *d*, cancel upon taking the ratio of two channels:

20 $$\begin{aligned} \frac{A_{\text{ blue }} }{A_{\text{ red }} }=\frac{\mu _{\text{ blue }} \,\,c\,\,d\,\,}{\mu _{\text{ red }} \;\;c\,\,d\,\,}=\frac{\mu _{\text{ blue }} }{\mu _{\text{ red }} } \end{aligned}$$

**Fig. 3:**
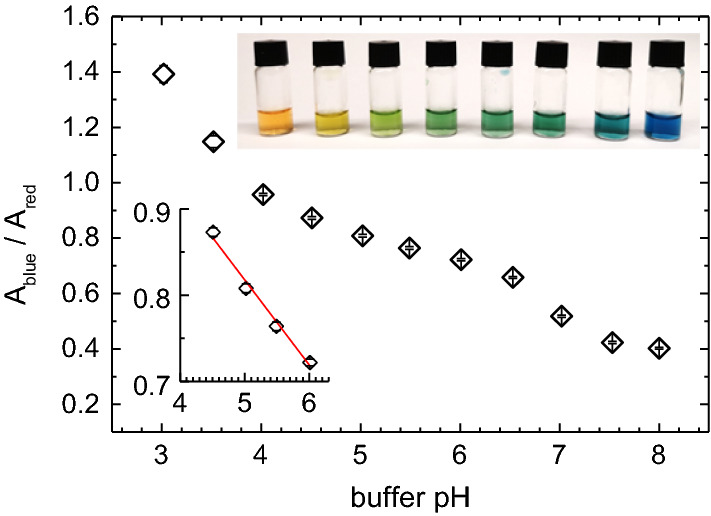
Testing micro-photometry. **a** Calibration curve displaying absorption ratio ${A}_{\mathrm{blue}}/{A}_{\mathrm{red}}$ for different pH. Upper inset: visual appearance of the indicator fluid in water buffered to different pH values ranging 3–8. Lower inset: close-up for the pH region of interest. The red line is a least square linear fit to the data

For calibration of absorbance ratios, we used the indicator mixture in buffer solutions adjusted to pH values between 1.9 and 8.9 in $0.50\pm 0.02~\hbox {pH}$ unit steps. Absorbance ratios evolve monotonously for each channel pair, with the best contrast for the green colour range provided by $A_{\mathrm{blue}}/A_{\mathrm{red}}$. We display the calibration curve for this pair in Fig. 3a, with the inset highlighting the pH range of interest (4.5 and 6.5). Note, that values range between 0.4 and 1.4, since no small numbers are involved in calculating the ratio from our transmission experiment. Absorbance ratios were stable in time and varied negligibly with position except for a small region very close to the outer cell walls, which we therefore excluded in evaluation. From repeated calibration measurements under varied illumination intensity, we infer a relative statistical uncertainty for the calibration points of $\Delta (A_{\mathrm{blue}}/A_{\mathrm{red}}) \sim 0.0015$ to 0.0032 slightly varying with pH. Using a linear fit function for the range of interest, this results in a residual uncertainty in pH of $\sim 0.02~\hbox {pH}$ units, corresponding to a gradient resolution of $\sim 0.02~\hbox {pH}$ units/$6~\upmu \hbox {m}$ for pumping and swimming experiments covering the pH interval between 4.5 and 6.5.

Depending on magnification and swimmer position in the field of view, we obtained observable excess concentration profiles with diameters in the micron range. Therefore, the far front pH was not always directly accessible. Then, the cell was shifted after the pH recordings to observe a pristine solvent region and the reference pH determined there.

Additional measurements using a sandwiched cell with two chambers filled with solutions of different pH demonstrated that, indeed, we record the average of local pH values and not the so-called column height, i.e. the total number of $\hbox {H}^{{+}}$ ions as integrated over the column of observation. The latter quantity is determined in fluorescence-based pH measurements, where it can be measured over a very restricted height interval, much smaller than any length scale of vertical pH change. Therefore, an exact determination of the pH at any given height is directly possible. Here, however, we vertically average local pH values over an extended height range

Three types of data are used: pH maps, collinear pH profiles and limiting slopes. For pH map design, a colour rendering developed in OriginLab 9.0®was employed, which also provided some additional graphical features. For reasons of clarity, we here reduce the pH resolution of the colour coding to intervals of 0.2 pH units. Further, the displayed pH maps are spatially resolved into $357\times 237$ blocks. At $10\times (5\hbox {x})$ magnification, each block covers an area of $5.8 \times 5.8~\upmu \hbox {m}^{{2}}$ ($11.6 \times 11.6\upmu \hbox {m}^{{2}}$) and the image size is $2072 \times 1375~\upmu \hbox {m}^{{2}}$ ($4144 \times 3750~\upmu \hbox {m}^{{2}}$). For the quantitative evaluation of profiles and slopes, of course, the full pH resolution is kept in the custom-written python scripts and fit routines.

## Results and discussion

### pH maps and shapes of the concentration field

**Fig. 4:**
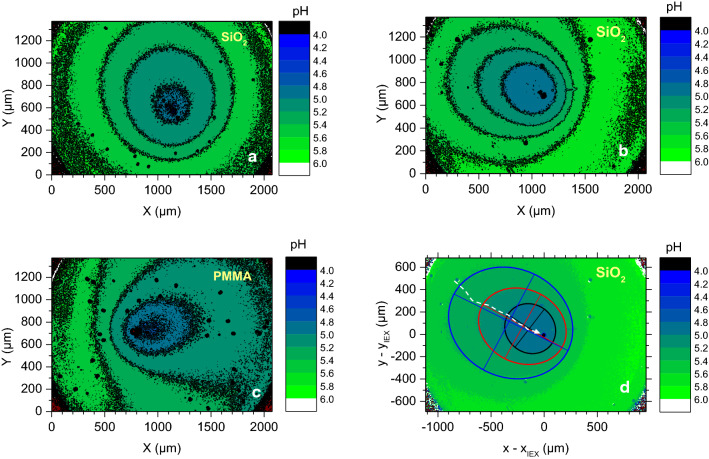
Exemplary pH maps for different swimmers taken at 10x magnification. The pH values are given in steps of 0.2 pH values indicated by the false colour rendered chart at the right. In **a**–**c** we also display the contours separating adjacent pH intervals. Note the grainy appearance in the transition region between different pH intervals resulting from small-scale local fluctuations in pH. **a** A slow swimmer ($\hbox {v} = 1.6~\upmu \hbox {m}~\hbox {s}^{{-1}}$) on glass, **b** a swimmer of moderate velocity ($\hbox {v} = 3.1~\upmu \hbox {m s}^{{-1}}$) on glass, and **c** a swimmer of moderate velocity ($\hbox {v} = 2.9~\upmu \hbox {m}~\hbox {s}^{{-1}}$) on PMMA. Note the approximately ellipse shaped iso-pH contours. Note further their characteristic dependence on substrate type and velocity. **d** PH map of the swimmer from **c** replotted in co-moving coordinates and with the trajectory added (white dashed line). The colours given in the key refer to ellipses fitted to iso-pH contours for $\hbox {pH} < 5.0$ (black), $\hbox {pH} < 5.2$ (red) and $\hbox {pH} < 5.4$ (blue). Crossed straight lines indicate major and minor axes and the overall orientation. The centres of the ellipses are shifted to the back of the IEX. Both distortion and shift get more pronounced at higher velocities

We start with a semi-quantitative analysis of height-averaged pH maps for different swimmers. Figure 4 shows the pH maps for single swimmers immersed in realistic salt-free water. Colour changes indicate the ongoing exchange of residual cations for protons, which diffuse outward. Since we plot absorbance ratios, the colour chart appears inverted as compared to the visual impression in the inset of Fig. 2, corresponding to transmission ratios. To cover a wide range of swimming velocities (1.5–5.2 $\upmu \hbox {m}~\hbox {s}^{{-1}}$), we prepared samples of different cargo number and used different size combinations $a_{\mathrm{cargo}}/a_{\mathrm{IEX}}$ for fine-tuning. In general, some 10–20% of the swimmers show sufficiently long and straight trajectories and, at the same time remained isolated from interference with neighbouring swimmers. As discussed in the ESI, section 3, only these fulfilled the selection criterion of an undisturbed pH field for quantitative measurements. In Fig. 4a–d, we give three examples: (a) a slow swimmer on glass ($v = 1.6~\upmu \hbox {m}~\hbox {s}^{{-1}}$), (b), (d) a swimmer of moderate velocity on glass ($\hbox {v} = 3.1~\upmu \hbox {m}~\hbox {s}^{{-1}})$ and (c) a swimmer of moderate velocity on PMMA ($v = 2.9~\upmu \hbox {m}~\hbox {s}^{{-1}}$). In Fig. 4a–c, the coordinate origin was placed at the lower left image corner, and we also display the iso-pH contours. In Fig. 4d we use IEX centred, co-moving coordinates and show the iso-pH contours approximated as ellipses.

The pH adjacent to the IEX surface was found to be between 4.95 and 5.15, and was systematically larger under swimming than for a stationary pump. The minimum pH always is located to the back of the IEX. The pH in the far field to the front was observed to be $\hbox {pH} \approx 5.5$ in all experiments, testifying propulsion into a pristine environment. The black contours clearly illustrate the approximately elliptic shape of pH contours. An elliptic distortion is readily suggested by simple geometric arguments, i.e. considering a section through the spatio-temporal cone of an iso-pH contour, increasingly inclined for increasing velocities (see Fig. S1 in the ESM). Closer inspection of our theoretical model shows that the shape is not expected to be precisely elliptical. Still, elliptical fits turned out to be a sufficient approximation for a semi-quantitative analysis of the shape of the iso-pH contours. With velocity increasing from (a)–(b), this distortion gets more pronounced. On the other hand, comparing Fig. 4b–c, ellipses appear much more “confined” on glass than on PMMA, indicating a larger diffusive spread normal to the propulsion direction for the latter case. In Fig. 4d, we again display the $\hbox {SiO}_{{2}}$ swimmer of moderate velocity ($\hbox {v} = 3.1~\upmu \hbox {m}~\hbox {s}^{{-1}}$), now using IEX centred, co-moving coordinates. We here include ellipses fitted to the contours separating different pH intervals. All three contours show that the long axis of the ellipses fitted to the iso-pH contours practically coincides with the propulsion direction, i.e. they are collinear with the trajectory. A first important finding therefore is a systematic deviation of the height-averaged profiles of linearly propelling swimmers from a circular shape; the concentration profiles take an approximately elliptical shape instead. The general shape of the concentration field persists at different velocities; it further is retained on the different substrates. Moreover, it demonstrates the coupling between propulsion velocity and solute diffusion as predicted by Eqs. (10) and (11).

Quantitative analysis of contour shapes was restricted to swimmers of constant number of cargo particles (i.e. velocity) showing straight trajectories at constant speed over several hundred microns and travelling in an undisturbed pristine environment of $\hbox {pH} \approx 5.5$. These can be found in the ESM in section 4. In general, we report the significant stability of the contour shapes of the swimmers, over extended periods. This attribute is crucial for the next step of systematically describe pH profiles.

### pH profiles

**Fig. 5:**
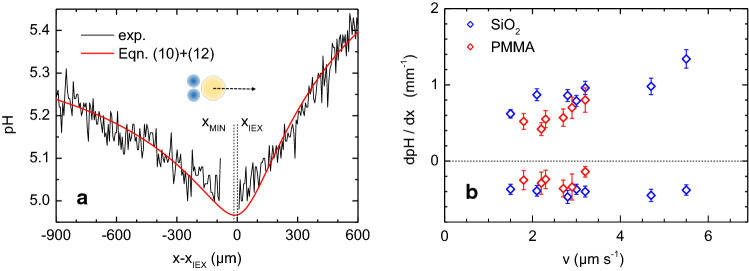
**a** Analysis of an experimental height-averaged pH profile recorded as collinear cross sections laid along $\mathbf{e} _{\mathrm{x}} \vert \vert \mathbf{v}$. As indicated, the dashed vertical lines denote the location of the minimum pH value and the IEX position as derived from the least square fit of Eq. (10) $+$ (12) (red solid line) to the data (black solid line). An excellent data description is achieved. The fit returns: $D = (1602\pm 24)~\upmu \hbox {m}^{{2}}~\hbox {s}^{{-1}}$, $\upmu ^{{+}} = (98.5\pm 5.1)~\hbox {molL}^{{-1}}~\hbox {s}^{{-1}}$, $\upmu ^{{-}} = 1.2\times 10^{{-3}}~\hbox {s}^{{-1}}$ and $c_{{\infty }} = (2.8\pm 0.1) \upmu \hbox {mol L}^{{-1}}$. **b** Front and back slopes of height-averaged pH profiles for swimmers of different velocities on different substrates as colour coded in the key. Gradient strengths were determined at a distance $x-x_{\mathrm{IEX}} =300~\upmu \hbox {m}$, i.e. close to the maximum values of the gradients. With the origin at $x_{\mathrm{IEX}}$, positive gradients correspond to the front slopes, and negative gradients to the back slopes

With the general shape of the measurable pH fields established, we next analyse the height-averaged pH profiles recorded as cross sections co-linear to the swimming direction. Figure 5 shows an exemplary profile recorded for a swimmer of moderate velocity $\hbox {v} = 2.8~\upmu \hbox {ms}^{{-1}}$ driven by two cargo particles. The experimental data show strong small-scale fluctuations, much larger than the statistical uncertainty of the photometric method. This effect was already visible in the iso-pH contours plotted in Fig. 3a–d. Such fluctuations are significantly reduced upon radial averaging, e.g. in experiments with stationary IEX (c.f. Fig. 7), and they are absent in samples without IEX resin added, e.g. in the buffered indicator solutions used for calibrating the pH. We therefore attribute this behaviour to the ongoing chemical reaction of the excess carbonic acid. Despite this scatter, the experimental data are well described by a least square fit of the height-averaged 3D diffusion model (Eq. (10) $+$ (12) with $H = 500~\upmu \hbox {m}$). The fit returns: $D = (1602\pm 24)~\upmu \hbox {m}^{{2}}~\hbox {s}^{{-1}}$, $\upmu ^{{+}} = (98.5\pm 5.1)~\hbox {mol}\,\hbox {L}^{{-1}}~\hbox {s}^{{-1}}$, $\upmu ^{{-}} = 1.2\times 10^{{-3}}~\hbox {s}^{{-1}}$ and $c_{{\infty }} = (2.8\pm 0.1) \upmu \hbox {mol}\, \hbox {L}^{{-1}}$. Here, the errors denote the standard error of the fit at a confidence level of 0.95.

We recorded such height-averaged pH profiles for selected swimmers propelling with different velocities on different substrate and fitted them to our model (for further examples see Figs. S10 and 11 in the ESM). All showed a number of characteristic features. Both to the front and the back of the IEX, the pH always appeared to increase approximately linearly with distance. With increasing velocity, the back slopes stay independent of velocity and the front profiles steepen. At the same time, the minimum concentration is located further to the back. The minima for swimmers propelling on $\hbox {SiO}_{{2}}$ show larger pH values for larger velocities. For both substrates, saturation effects set in at distances of some hundred microns. With increasing velocity, the onset of these saturation effects occurs closer to the front of the IEX position. Interestingly, the location of the steepest pH gradient appeared to be independent of velocity and substrate choice at $x-x_{\mathrm{IEX}} \approx 300~\upmu \hbox {m}$ for $\hbox {H} = 500~\upmu \hbox {m}$. Slopes determined at this distance are shown in Fig. 5b. The shown slopes qualitatively reproduce the analytical expectation from Eq. (14) (see Fig. 2d).

To test the implementation of the corrections for finite cell height and of height averaging, we also performed experiments in cells of different heights. These nicely reproduced the above discussed general features. For cells with smaller height, the profiles generally became much steeper and the minima became more pronounced (See Fig. S11d in the ESI). Moreover, the location of the steepest slopes, however, shifted to $x-x_{\mathrm{IEX}} \approx 140~\upmu \hbox {m}$. Again, all profiles were well described by our model, and the fit parameters obtained did not vary upon altering the cell height.

**Fig. 6:**
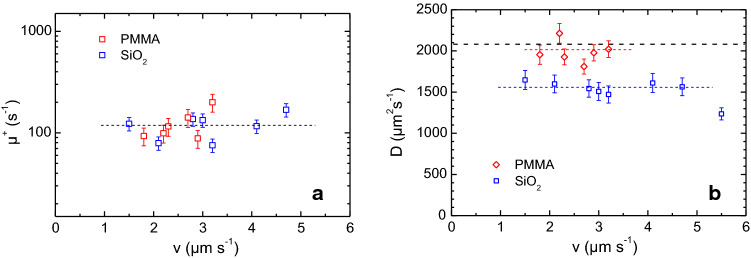
Fit parameters as a function of velocity, as obtained from fits of Eq. (10) $+$ (12). Different symbol colour denotes swimmers propelling on two different substrates: PMMA (red) and $\hbox {SiO}_{{2}}$ (blue). Error bars denote the standard error of the fitted parameters at a confidence level of 0.95. **a** Production rates $\upmu ^{{+}}$ determined different swimmers. The averaged value is marked by the dashed line at $\upmu ^{{+}} = (98.5\pm 5.1)~\hbox {molL}^{{-1}}~\hbox {s}^{{-1}}$. **b** Diffusion coefficients. The dashed black line indicates Nernst’s mutual Diffusion coefficient $\hbox {D} = 2096~\upmu \hbox {m}^{{2}}~\hbox {s}^{{-1}}$ for carbonic acid. The averaged values for the two substrates are $D_{\mathrm{PMMA}} = (2002\pm 132)~\upmu \hbox {m}^{{2}}~\hbox {s}^{{-1}}$ (red dashed line) and $D_{\mathrm{SiO2}} = (1585\pm 142)~\upmu \hbox {m}^{{2}}~\hbox {s}^{{-1}}$ (blue dashed line)

We now turn to the main fit results. These are given in terms of averages over several selected swimmers of same IEX/PS combination, substrate, and velocity. For all swimmers, the production rates agree well within experimental scatter. The average value of $\upmu ^{{+}} = (98.5\pm 13)~\hbox {mol L}^{{-1}}~\hbox {s}^{{-1}}$ is independent of velocity and substrate material. It is displayed in Fig. 6a. Here, the error denotes the standard deviation of the averages. The relatively large statistical scatter of individual data points taken at different velocities appeared to be correlated to the corresponding IEX sizes as determined from the microscopic images of the swimmers. The average loss rate was determined to be $\upmu ^{{-}} = (1.2\, \pm \,0.6) 10^{{-3}}~\hbox {s}^{{-1}}$, again independent of velocity, substrate choice and cell height. Here, the error denotes the standard deviation of the averages. The average production rates are reasonable for a high efficiency commercial IEX. The average loss rates as well as the background electrolyte concentration are consistent with the reaction constants in the assumed chemical reaction scheme (Eq. (18) [67]) and the adjusted realistic salt-free conditions [4].

Since the loss rate is small, diffusion dominates the decay of the excess concentration. Figure 6b displays fitted diffusion coefficients for swimmers of different velocities on the two substrates. For each substrate, the data display some statistical variation between individual swimmers, but no systematic trend as a function of velocity. The decrease observed for the fastest swimmer on SiO2 can be attributed to incomplete coverage of the elongated pH fields by the observable area. We thus exclude this point at $\hbox {v} = 5.5~\upmu \hbox {ms}^{{-1}}$ in the averaging of the fit results. On PMMA, the average diffusion coefficient is $D_{\mathrm{PMMA}} = (2002\pm 132)~\upmu \hbox {m}^{{2}}~\hbox {s}^{{-1}}$, where the error again denotes the standard deviation. This value is only slightly smaller than and, within statistical uncertainty, fully consistent with the Nernst expectation value $D_{\mathrm{Nernst}} = 2096~\upmu \hbox {m}^{{2}}~\hbox {s}^{{-1}}$ (dashed black line). By contrast, for $\hbox {SiO}_{{2}}$, we obtain an average diffusion coefficient of $D_{\mathrm{SiO2}} = (1585\pm 142)~\upmu \hbox {m}^{{2}}~\hbox {s}^{{-1}}$. The diffusion coefficient for swimmers on $\hbox {SiO}_{{2}}$ substrates is significantly lower than the expected one. Such a pronounced impact of the substrate type on the found values of the diffusion coefficient is not expected from our model, which neglects the impact of inhomogeneous solvent advection on the diffusive dispersion of chemicals.

### Electro-osmotic pumping on different substrates

**Fig. 7:**
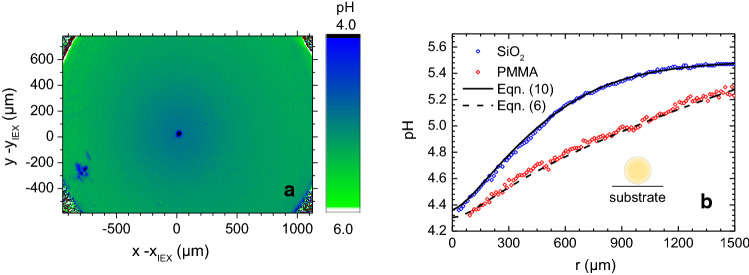
Complementary pumping experiment. **a** pH map for a stationary pump on $\hbox {SiO}_{{2}}$ with IEX centred coordinates recorded at $t = 200~\hbox {s}$. **b** radially averaged pH profiles for two the different substrates, as indicated. The profile for the more highly charged $\hbox {SiO}_{{2}}$ substrate displays the steeper slope. Note also the lower central pH values as compared to those of swimmers. As compared to Fig. 5a, the statistical scatter is considerably reduced by the radial averaging. The dashed and the solid lines represent least square one parameter fits of our model to the data. For details, see text

To test the above suggestion, we performed an additional experiment under chemically identical conditions, but now with the IEX fixed to the substrate and no added cargo, following the protocol established by Niu et al. [61]. That is, here, ion exchange is to drive a micro-fluidic pump. It is known from the literature that in this case, any unidirectional solvent current is absent. However, there is a centrally converging electro-osmotic flow of velocity $\hbox {v}_{\mathrm{eo}}$ along the substrate driven by the diffusio-electric field induced by the pH gradient [57, 61]. In closed cells, it leads to the formation of a complex toroidal, but stationary advection pattern throughout the complete cell [59]. Theory expects $\hbox {v}_{\mathrm{eo}}$ to be directly proportional to the substrate zeta potential [2]. Recently, we have confirmed this experimentally for the two substrates addressed in the present paper [58]. Pumps are therefore well suited to test the influence of substrate zeta potential on the shape of the pH field. To first approximation, a feedback of the electro-osmotically driven, convergent solvent advection on the pH map should show up as a substrate-dependent, radially symmetric steepening of gradients. A typical pH map obtained on $\hbox {SiO}_{{2}}$ is shown in Fig. 7a. In good agreement with previous observations, the pH maps are centro-symmetric and evolve slowly over time, i.e. in line with Eqs.  (5) and (6), no stationary state exists [57]. Figure 7b displays the radially averaged pH maps for $t = 200~\hbox {s}$.

The pH map shows a radially symmetric pH increase. Due to radial averaging, the profiles appear smoother than those of swimmers, and due to the centrally fixed IEX, they can be measured over larger distances. As before, finite height and the projection correction are decisive, to rationalize the shallowness of the minima of the pH field. Minimum pH values are somewhat lower than for swimmers. As before in the swimming experiments on the different substrates, we here had the same chemical composition, the same type of IEX, the same cell geometry and the same background electrolyte concentration for the pumps working on different substrates. In the absence of a feedback between electro-osmotically driven solvent convection and dispersion of chemicals, we would thus expect identical slopes of the pH profiles. However, we observe a pronounced steepening of the pH profile for $\hbox {SiO}_{{2}}$. As in our experiments with swimmers, the observation of a steeper slope for $\hbox {SiO}_{{2}}$, is consistent with its larger zeta potential. We conclude that in both our pumping and swimming experiments, the steepening of the gradients relates to the substrate induced electro-osmotic solvent advection.

For a quantitative evaluation, we here use adaptations of our model to the pump specific circumstances. For PMMA, the dashed line represents a single parameter fit using Eq. (6) $+$ (12). Here, we fixed the inputs to the averages obtained from the swimmer experiments on PMMA: $v = 0~\upmu \hbox {ms}^{{-1}}$, $D = D_{\mathrm{PMMA}} = 2002~\upmu \hbox {m}^{{2}}~\hbox {s}^{{-1}}$, and $\upmu ^{{-}} =1.2\times 10^{{-3}}~\hbox {s}^{{-1}}$. We further equated *r* to $\vert x\vert$, set $t = 200~\hbox {s}$ corresponding to the time at which the pH map was recorded and fixed, and we fixed, $c_{{\infty }} = 3.24~\hbox {mol L}^{{-1}}$ to the experimentally determined far field value. The production rate was left as free parameter to account for the variations in IEX size. For this quantity we here obtain $\upmu ^{{+}} = (153\pm 12)~\hbox {mol L}^{{-1}}~\hbox {s}^{{-1}}$. The excellent fit quality demonstrates, the applicability of our model for electro-osmotic pumping, in the case of PMMA. In this case, the swimmer experiments had shown that the coupling between electro-osmotically driven solvent flow and dispersion of the chemical is negligibly small.

By contrast, this approach using $v = 0$ does hold for $\hbox {SiO}_{{2}}$ substrates. A one parameter fit with D$_{\mathrm{SiO2}} = 1585~\upmu \hbox {m}^{{2}}~\hbox {s}^{{-1}}$ and the other input parameters fixed as before fails qualitatively to describe the data (Fig. S12 in the ESI). Alternatively, we tried to fit the $\hbox {SiO}_{{2}}$ data using a two parameter least-square fit of our model using Eqs. (9) and (10) in Eq. (12) restricted to $x > 0$, i.e. the front profiles. We now fixed $D = \hbox {D}_{\mathrm{SiO2}} = 1585~\upmu \hbox {m}^{{2 }}~\hbox {s}^{{-1}}$, $\upmu ^{{-}} =1.2\times 10^{{-3}}~\hbox {s}^{{-1}}$, $t = 200~\hbox {s}$, and $c_{{\infty }} = 3.1~\hbox {mol L}^{{-1}}$. Production rate and effective velocity were left as free parameters, the first mainly setting the depth of the minimum and the second determining the profile shape. The fit is shown as black line in Fig. 7b. It excellently describes the data. We obtain a production rate of $\upmu ^{{+}} = 127\pm 10~\hbox {mol L}^{{-1}}~\hbox {s}^{{-1}}$ and an effective velocity of $v_{\mathrm{eff}} = 4.1~\upmu \hbox {m}~\hbox {s}^{{-1}}$. The production rate is close to the average value obtained above. The effective velocity is in the range of swimmer velocities for swimmers with several cargo particles on $\hbox {SiO}_{{2}}$. The corresponding Peclet number is thus similar to those of swimmers.

### Feedback mechanisms

Generally, fits of our model to the data were of high quality, and they excellently describe the shape of pH profiles taken in swimming direction. For swimmers propelling on PMMA, average production rates are reasonable for a high efficiency commercial IEX, loss rates and equilibrium proton concentrations are fully consistent with the proposed chemical reaction scheme and the average diffusion coefficients agree with the expectation of Nernst’s mutual diffusion coefficient. The same observations were made for pumps working on PMMA. The same fit quality is seen on $\hbox {SiO}_{{2}}$ substrates. Further, the same production and loss rates are obtained within experimental scatter. However, on this more highly charged substrate fitted diffusion coefficients now are significantly smaller than the expectation value. Quite remarkably, also the experiments performed with electro-osmotic pumps on the two different substrates are very well captured by suitable modifications of the model corresponding to the different experimental geometry. As before, correct implementation of the effects of restricted geometry and of height averaging turned out to be decisive for a correct description. Both measures are also crucial to capture the cell height dependence of measured gradients.

From the extensive comparison of high quality experimental data to our model, we are for the first time able to demonstrate and quantify the feedback mechanisms between substrate induced inhomogeneous solvent flows and the diffusive dispersion of chemicals shaping the driving gradients. While the model does not explicitly account for such flows, it can accommodate them for swimmers of a given velocity *via* a single parameter, i.e. a reduced effective diffusion coefficient. For pumps, only one additional free parameter is needed to account for the height-averaged net inflow of solvent along the substrate in terms of an effective velocity.

This treatment of spatially inhomogeneous solvent flows worked well for the moderate velocities involved in the present experiments. In our approach, the effective velocity corresponds to average of height-dependent solvent weighted by the height-dependent concentration distribution. In the present case, it is negative. That is, we have a net advective inflow of chemicals. The dispersion of carbonic acid is thus modified by advection, although diffusion still dominates. This may not hold any longer, if larger velocities are involved or IEX beads with larger production rates are used in the pump experiments. In fact, preliminary pump experiments using mm-sized epoxy resin-based IEX reveal the emergence of a region with strictly linear, but very slow outward pH increase extending a few hundred microns. Then a steeper increase—similar to the ones observed here—sets in. Such profile shapes are qualitatively incompatible with our model. Clearly, a full hydrodynamic modelling of the solvent flow is necessary in such cases (but of course already highly desirable also at moderate velocities).

Our investigations also return useful information on the other feedback mechanisms involved in micro-swimming. For instance, they confirm the known dependence of pH gradients on production rate. For the first time they demonstrate and quantify the coupling between swimmer speed and gradient steepness. On PMMA the coupling is independent of the additional but slow inhomogeneous solvent advection. On $\hbox {SiO}_{{2}}$ we observe a modification which does not alter the functional dependence, but its magnitude. As discussed, the presumably complex solvent flow pattern [61] is successfully mapped onto a reduced effective diffusivity. Our study also reveals the influence of loss rates on this coupling. With $\upmu ^{{-}} = 1.2\times 10^{{-3}}~\hbox {s}^{{-1}}$, these effects are already discernible, but still very small, such that in the present experiments diffusion still dominates gradient shaping.

### Implications

We observed that the gradients occurring at the cell walls (the ground slopes) behave qualitatively similar to the height-averaged gradients. They further extend over much larger distances. This allows to discuss some important consequences of the presence of charge bearing substrates for micro-fluidic experiments in general and micro-swimming in particular. First, the ground slopes to the back of a swimmer are only weakly dependent on swimmer velocity, even in the case of finite loss rates. This confirms the assumption of a constant diffusio-electric field at the cargo location, which previously was made to explain the increase of swimmer velocities in equidistant steps upon stepwise increasing the number of cargo particles (c.f. [54] and Eqs. S1 and S2 in the ESI). Moreover, the remaining velocity dependence (c.f. Fig. S4 in the ESI) is qualitatively consistent with the experimentally observed slight decrease in the step size for larger velocities and the apparently too large step size as the first cargo particle becomes trapped (c.f. [53] and Fig. S5 of the ESI). Our model further elegantly explains the observed slower velocities for swimmers propelling on PMMA as compared to those surfing $\hbox {SiO}_{{2}}$ substrates under otherwise identical boundary conditions [53]. Since the feedback between the electro-osmotic flows and the effective diffusion coefficient of the chemical is more pronounced for substrates with larger zeta potential, the ground slopes are steeper for $\hbox {SiO}_{{2}}$ due to their dependence on effective diffusivity at finite $\upmu ^{{-}}$ (c.f. section 2 in the ESI). Consequently, the cargo particles exert larger propelling flows on the IEX in the case of the enhanced ground slope on $\hbox {SiO}_{{2}}$.

Second, we expect charged substrates to interfere with the transport of dissolved chemicals in porous media, like sand beds or withered rocks as well as in many micro-fluidic applications. The resulting inhomogeneous solvent flows may, for example, explain observations of unexpectedly large proton diffusion coefficients some recent mixing experiments [45, 69]. There, the authors joined two chemicals in a y-junction with rectangular cross section, fabricated on quartz glass by a wet-etching technique [70]. Using confocal fluorescence microscopy, they measured the horizontal pH profiles at mid-cell height in the downstream arm as a function of distance from the junction. For the mixing of HCl with water, they found a much *too large* proton diffusivity. The effect was more pronounced in the case of mixing HCl with NaOH. It may be rationalized in the light of the present findings: The presence of a cross-cell gradient in pH will trigger electro-osmotic flows across the bottom and top walls towards the HCl-rich side of the channel. Solvent incompressibility then enforces the formation of a counter flow at mid-cell height, where the pH was determined. This additional flow is more pronounced for a larger pH difference between the two incoming fluids. It enhances the spreading of chemicals in the gradient direction resulting in a flattened gradient at mid-cell height. In the cases investigated above, the solvent flows retarded the spreading and, consequently, steepened gradients were observed. The underlying coupling mechanism, however, appears to be the same in both types of experiment. We currently revisit the mixing experiments using three channel photometry and different channel materials for the micro-fluidic junction.

Finally, our observations may be relevant for chemo-taxis. Diffusio-phoresis-based chemo-taxis [8, 10] in the bulk is well known for molecular, macromolecular and particulate systems [17, 56]. It finds application in latex deposition or particle accumulation in pores [71]. It further appears to be the underlying condition for (logarithmic) sensing and orientation of artificial and biological entities like macromolecules, bacteria or sperms [11, 12]. Based on the existence of such long ranged gradients, nature evolved an exquisite sensitivity of the signalling pathways in biological systems. For example, for sperms, already minute concentration differences of a few ions of $\hbox {Ca}^{{++}}$ and $\hbox {H}^{{+}}$ along the sperm contour trigger a precise reaction of the sperm’s flagellum to the corresponding gradients [19]. The logarithmic dependence on concentration gradients can be exploited in an alternative way by introducing electro-osmotic solvent convection along ta charged substrate, This has been realized for 2D particle assembly in many experiments or the stabilization of the trails of expelled surplus cargo forming in the back of a swimmer [25]. Moreover, it also opens the possibility for mutual swimmer–swimmer interactions via diffusio-phoretic effects. These have already been seen in different experiments [26], but their potential for long-ranged mutual swimmer–swimmer interactions has not been addressed in systematic systematic studies combining swimmer tracking with simultaneous gradient determination. Further, modular and other swimmers may be guided by the flow fields generated by combinations of different electro-osmotic pumps. The current observations and our model may help in realizing such interesting future experiments.

## Conclusions

Using three-channel micro-photometry with an improved pH resolution of 0.02 pH values per $6\upmu \hbox {m}$, we determined height-averaged pH fields forming around IEX beads either moving as modular micro-swimmer or staying at a fixed location in the case of electro-osmotic pumps. For the first time, this enabled an extensive *in situ* characterization of driving chemical fields for modular swimmers. Gradients here were formed by the continuous production of carbonic acid, which as a simple 1:1 electrolyte is known to display Nernst-type mutual diffusion, but at the same time is known to couple to the dissociation equilibrium of $\hbox {CO}_{2}$. Moreover, identical conditions of realistically salt-free systems were used in all experiments, while two different substrates differing in their zeta potential were studied. Consistent with previous experiments the pH fields of pumps were found to be spherically symmetric. For the swimmers, the pH maps showed a characteristic elliptical elongation of the pH field and the presence of pronounced small-scale fluctuations. pH profiles taken in swimming direction were asymmetric with steeper pH gradients at the swimmer front. pH gradients forming at swimmers and pumps on $\hbox {SiO}_{{2}}$ were observed to be systematically larger than those forming on PMMA. On both substrates, swimmer front slopes steepened with increasing velocity, while the back slopes stayed independent of swimmer speed.

For modelling we adapted known solutions of the three-dimensional diffusion equation for a 1:1 electrolyte to the specific circumstances of our experiments. Our model accounts for a point source producing carbonic acid in ion exchange reactions. It incorporates loss rates and equilibrium concentrations as determined by the chemical reaction scheme appropriate for carbonic acid in otherwise salt-free water as well as for the diffusive dispersion of this chemical in the presence of a spatially homogeneous solvent flow of constant velocity. We further included effects of finite cell height and projection effects arising from height averaging in the photometric experiments. It is important to note, however, that the dispersion of chemicals by inhomogeneous advection as driven by electro-osmotic flows along the substrate is not yet explicitly considered.

From the comparison of our model to the experimental data, we obtain estimates of a number of relevant quantities. Production and loss rates for carbonic acid are found to be independent of swimming speed and choice of substrate. The effective diffusion coefficient is close to the Nernst expectation value for the weakly charged substrate. For the more highly charged substrate, we observe steeper than expected gradients, and the fits return a reduced effective diffusivity.

From this and complementary experiments on stationary IEX pumps, we finally can characterize the gradient shaping processes present in our systems. For the first time we can provide clear evidence of a formerly not well characterized significant feedback mechanism between the diffusive dispersion of chemicals forming the concentration gradient and a gradient induced solvent convection modifying the dispersion. Further careful analysis allowed its unequivocal identification: Large-scale diffusion of simple 1:1 electrolytes is modified by gradient-driven electro-osmotic solvent flows along the differently charged substrates. This feedback is weak on weakly charged substrates but gains importance for the more highly charged $\hbox {SiO}_{{2}}$ substrate. Remarkably, and despite the presumably complex solvent flow pattern resulting from this effect, its influence on the gradient shaping can be absorbed into an effective diffusion coefficient, even within the simplifying assumptions still underlying the present model. This effect is also present in pumps on $\hbox {SiO}_{{2}}$, and could be accounted for in our model in an approximative way. Further work aiming at a quantification based on the full solvent flow field is in progress.

We further discussed the relevance of this feedback for recent and future work in micro-fluidics and micro-swimming. With many challenges remaining, we anticipate our study may advance the scope of quantitative modelling and targeted application of diffusion-phoretic flows in general and of phoretic swimming in particular.

## Supplementary Information

Below is the link to the electronic supplementary material.

Supplementary material 1 (docx 5733 KB)

## Data Availability

This manuscript has associated data in a data repository. [Authors’ comment: The data used to support the findings of this study are available from the corresponding author upon request.]

## References

[1] E.M. Purcell, Am. J. Phys. **45**, 3–11 (1977). 10.1119/1.10903

[2] J.L. Anderson, Ann. Rev. Fluid Mech. **21**, 61–99 (1989). 10.1146/annurev.fl.21.010189.000425

[3] Á.V. Delgado, F. Carrique, R. Roa, E. Ruiz-Reina, Curr. Opin. Colloid Interface Sci. **24**, 32–43 (2016). 10.1063/5.0010692

[4] Botin D, Carrique F, Ruiz-Reina E, Palberg T (2020). J. Chem Phys..

[5] A. Würger, Rep. Prog. Phys. **73**, 126601 (2010). 10.1088/0034-4885/73/12/126601

[6] F.M. Weinert, C.B. Mast, D. Braun, Phys. Chem. Chem. Phys. **13**, 9918–9928 (2011). 10.1039/C0CP02359K 10.1039/c0cp02359k 21240434

[7] H. Jiang, N. Yoshinaga, M. Sano, Phys. Rev. Lett. **105**, 268302 (2010). 10.1103/PhysRevLett.105.268302 10.1103/PhysRevLett.105.268302 21231718

[8] J.L. Anderson, D.C. Prieve, Sep. Purific. Rev. **13**, 67–103 (1984). 10.1080/03602548408068407

[9] D. Velegol, A. Garg, R. Guha, A. Kar, M. Kumar, Soft Matter **12**, 4686–4703 (2016). 10.1039/C6SM00052E 10.1039/c6sm00052e 27174044

[10] P.O. Staffeld, J.A. Quinn, J. Colloid Interface Sci. **13**, 69–87 (1989). 10.1016/0021-9797(89)90079-9

[11] J. Palacci, B. Abécassis, C. Cottin-Bizonne, C. Ybert, L. Bocquet, Phys. Rev. Lett. **10**, 138302 (2010). 10.1103/PhysRevLett.104.138302 10.1103/PhysRevLett.104.138302 20481918

[12] J. Palacci, B. Abécassis, C. Cottin-Bizonne, C. Ybert, L. Bocquet, 980–994 (2010). 10.1039/C1SM06395B 10.1039/c1sm06395b 41804027

[13] T.-Y. Chang, D. Velegol, J. Colloid Interface Sci. **424**, 120–123 (2014). 10.1016/j.jcis.2014.03.003 10.1016/j.jcis.2014.03.003 24767507

[14] R. Niu, T. Palberg, Soft Matter **14**, 3435–3442 (2018). 10.1039/C8SM00256H 10.1039/c8sm00256h 29589860

[15] S. Battat, J.T. Ault, S. Khodaparast, H.A. Stone, Soft Matter **15**, 3879–3885 (2019). 10.1039/C9SM00427K 10.1039/c9sm00427k 31021341

[16] M.J. Esplandiu, D. Reguera, J. Fraxeidas, Soft Matter **16**, 3717–3726 (2020). 10.1039/D0SM00170H 10.1039/d0sm00170h 32232286

[17] J.M. Schurr, B.S. Fujimoto, L. Huynh, D.T. Chiu, J. Phys. Chem. B **117**, 7626–7652 (2013). 10.1021/jp302587d 10.1021/jp302587d 23656252

[18] H. Stark, Acc. Chem. Res. **51**, 2681 (2018). 10.1021/acs.accounts.8b00259

[19] U.B. Kaub, L. Alvarez, Eur. Phys. J. Spec. Top. **225**, 2119–2139 (2016). 10.1140/epjst/e2016-60097-1

[20] Singh DP, Choudhury U, Fischer P, Mark AG (2017). Adv. Mater..

[21] A. Aubret, M. Youssef, S. Sacanna, J. Palacci, Nat. Phys. **14**, 1114–1118 (2018). 10.1038/s41567-018-0227-4

[22] T. Speck, Soft Matter **16**, 2652–2663 (2020). 10.1039/D0SM00176G

[23] S. Heckel, J. Grauer, M. Semmler, T. Gemming, H. Löwen, B. Liebchen, J. Simmchen, *Langmuir* (2020). 10.1021/acs.langmuir.0c01568 10.1021/acs.langmuir.0c01568 32825804

[24] J.L. Moran, P.M. Wheat, J.D. Posner, Phys. Rev. E **81**, 065302(R) (2010). 10.1103/PhysRevE.81.065302

[25] A. Reinmüller, H.J. Schöpe, T. Palberg, Langmuir **29**, 1738–1742 (2013). 10.1021/la3046466 10.1021/la3046466 23343457

[26] W. Duan, W. Wang, S. Das, V. Yadav, T.E. Mallouk, A. Sen, Annu. Rev. Anal. Chem. **8**, 311–333 (2015). 10.1146/annurev-anchem-071114-040125 10.1146/annurev-anchem-071114-040125 26132348

[27] J.L. Moran, J.D. Posner, Annu. Rev. Fluid Mech. **49**, 511–540 (2017). 10.1146/annurev-fluid-122414-034456

[28] C. Kurzthaler, C. Devailly, J. Arlt, T. Franosch, W.C.K. Poon, V.A. Martinez, A.T. Brown, Phys. Rev. Lett. **121**, 078001 (2018). 10.1103/PhysRevLett.121.078001 10.1103/PhysRevLett.121.078001 30169062

[29] C. Zhou, H.P. Zhang, J. Tang, W. Wang, Langmuir **34**, 3289–3295 (2018). 10.1021/acs.langmuir.7b04301 10.1021/acs.langmuir.7b04301 29436833

[30] R. Niu, A. Fischer, T. Palberg, T. Speck, ACS Nano **12**, 10932–10938 (2018). 10.1021/acsnano.8b04221 10.1021/acsnano.8b04221 30346687

[31] X. Chen, C. Zhou, W. Wang, Chem. Asian J. **14**, 2388–2405 (2019). 10.1002/asia.201900377

[32] T. Speck, Phys. Rev. E **99**, 060602(R) (2019). 10.1103/PhysRevE.99.060602

[33] T. Speck, J. Tallieur, J. Palacci, New J. Phys. **22**, 060201 (2020). 10.1088/1367-2630/ab90d9

[34] R. Kapral, J. Chem. Phys. **138**, 020901 (2013). 10.1063/1.4773981

[35] A. Altemose, M.A. Sánchez-Farrán, W. Duan, S. Schulz, A. Borhan, V.H. Crespi, A. Sen, Angew. Chem. Int. Ed. **56**, 7817–7821 (2017). 10.1002/anie.201703239 10.1002/anie.201703239 28493638

[36] C. Zhou, X. Chen, Z. Han, W. Wang, ACS Nano **13**, 4064–4072 (2019). 10.1021/acsnano.9b08421 10.1021/acsnano.8b08276 30916919

[37] J. Palacci, S. Sacanna, A. Vatchinsky, P.M. Chaikin, D.J. Pine, J. Am. Chem. Soc. **135**, 15978–15981 (2013). 10.1021/ja406090s 10.1021/ja406090s 24131488

[38] D. Feldmann, S.R. Maduar, M. Santer, N. Lomadze, O.I. Vinogradova, S. Santer, Sci. Rep. **6**, 36443 (2016). 10.1038/srep36443 10.1038/srep36443 PMC5093767 27808170

[39] F.A. Lavergne, H. Wendehenne, T. Bäuerle, C. Bechinger, Science **364**, 70–74 (2019). 10.1126/science.aau5347 10.1126/science.aau5347 30948548

[40] Sachs J, Kottapalli SN, Fischer P, Botin D, Palberg T (2020). Colloid Polym. Sci..

[41] Fernandez-Rodriguez MA, Grillo F, Alvarez L, Alvarez L, Rathlef M, Buttinoni I, Volpe G, Isa L (2020). Nat. Commun..

[42] C.C. Overly, K.D. Lee, E. Berthiaume, P.J. Hollenbeck, Proc. Natl. Am. Soc. **92**, 3156–3160 (1995). 10.1523/JNEUROSCI.16-19-06056.1996

[43] J. Diao, L. Young, S. Kim, E. Fogarty, S. Heilman, P. Zhou, M. Shuler, M. Wu, M. DeLisa, Lab Chip **6**, 381–388 (2006). 10.1039/B511958H 10.1039/b511958h 16511621

[44] S. Krag Gjetting, C. Karkov Ytting, A. Schulz, A. Thoe Fuglsang, *J. Exp. Botany* **63**, 3207-3218 (2012). 10.1093/jxb/ers040 10.1093/jxb/ers040 PMC3350929 22407646

[45] K. Shinohara, Y. Sugii, K. Okamoto, H. Madarame, A. Hibara, M. Tokeshi, T. Kitamori, Meas. Sci. Technol. **15**, 955–960 (2004). 10.1088/0957-0233/15/5/025

[46] F.M. Möller, F. Kriegel, M. Kieß, V. Sojo, D. Braun, Angew. Chem. Int. Ed. **56**, 2340–2344 (2017). 10.1002/anie.201610781 10.1002/anie.201610781 28117546

[47] C. Wu, J. Dai, X. Li, L. Gao, J. Wang, J. Liu, J. Zheng, X. Zhan, J. Chen, X. Cheng, M. Yang, J. Tang, Nat. Nanotechnol. (2020). 10.1038/s41565-020-00825-9 10.1038/s41565-020-00825-9 33432205

[48] Y. Avnir, Y. Barenholz, Anal. Biochem. **347**, 34–41 (2005). 10.1016/j.ab.2005.09.026 10.1016/j.ab.2005.09.026 16289011

[49] J. Isaksson, D. Nilsson, P. Kjäll, N.D. Robinson, A. Richter-Dahlfors, M. Berggren, Organ. Electron. **9**, 303–309 (2008). 10.1016/j.orgel.2007.11.011 10.1038/nmat1963 17643105

[50] U.B. Kaupp, N.D. Kashikar, I. Weyand, Annu. Rev. Physiol. **70**, 93 (2008). 10.1146/annurev.physiol.70.113006.100654 10.1146/annurev.physiol.70.113006.100654 17988206

[51] B. Liebchen, H. Löwen, Acc. Chem. Res. **51**, 2982 (2018). 10.1021/acs.accounts.8b00215 10.1021/acs.accounts.8b00215 30375857

[52] R. Niu, T. Palberg, Soft Matter **14**, 7554–7568 (2018). 10.1039/C8SM00995C 10.1039/c8sm00995c 30073235

[53] R. Niu, D. Botin, A. Reinmüller, T. Palberg, Langmuir **33**, 3450–3457 (2017). 10.1021/acs.langmuir.7b00288 10.1021/acs.langmuir.7b00288 28346787

[54] B. Liebchen, R. Niu, T. Palberg, H. Löwen, Phys. Rev. E **98**, 052610 (2018). 10.1103/PhysRevE.98.052610

[55] Liebchen B, Marenduzzo D, Pagonabarraga I, Cates ME (2015). Phys. Rev. Lett..

[56] B. Liebchen, H. Löwen, in K. Lindenberg, R. Metzler, G. Oshanin (eds.) *Chemical Kinetics Beyond the Textbook* (World Scientific, 2019) pp. 493-516

[57] R. Niu, S. Khodorov, J. Weber, A. Reinmüller, T. Palberg, NJP **19**, 115014 (2017). arXiv:1708.02003

[58] D. Botin, J. Wenzel, R. Niu, T. Palberg, Soft Matter **14**, 8191–8204 (2018). 10.1039/C8SM00934A 10.1039/c8sm00934a 30259053

[59] A.A. Farniya, M.J. Esplandiu, D. Reguera, A. Bachtold, Phys. Rev. Lett. **111**, 168301 (2013). 10.1103/PhysRevLett.111.168301 10.1103/PhysRevLett.111.168301 24182306

[60] M.J. Esplandiu, A.A. Farniya, D. Reguera, J. Chem. Phys. **144**, 124702 (2016). 10.1063/1.4944319 10.1063/1.4944319 27036470

[61] R. Niu, P. Kreissl, A.T. Brown, G. Rempfer, D. Botin, C. Holm, T. Palberg, J. de Graaf, Soft Matter **13**, 1505–1518 (2017). 10.1039/C6SM02240E 10.1039/c6sm02240e 28127614

[62] J. Bodin, Water Resour. Res. **51**, 1860–1871 (2015). 10.1002/2014WR015910

[63] M. Heidari, A. Bregulla, S. Muinos Landin, F. Cichos, R. von Klitzing, Langmuir **36**, 7775 (2020). 10.1021/acs.langmuir.0c00461 10.1021/acs.langmuir.0c00461 32544339

[64] F.J. Millero, Geochim. Cosmochim. Acta. **59**, 661–677 (1995). 10.1016/0016-7037(94)00354-O

[65] F.J. Millero, R. Feistel, D.G. Wright, T.J. McDougall, Deep-Sea Res. **I**(55), 50–72 (2008). 10.1016/j.dsr.2007.10.001

[66] E. Ruiz-Reina, F. Carrique, J. Phys. Chem. B **112**, 11960–11967 (2008). 10.1021/jp8027885 10.1021/jp8027885 18767775

[67] P. Vanysek, *CRC Handbook of Chemistry and Physics*, 83rd edn. CRC Press, Boca Raton, pp. 76–78 (2002). 10.1016/j.gca.2011.02.010

[68] R.E. Zeebe, Geochim. Cosmochim. Acta **75**, 2483–2498 (2011). 10.1016/j.gca.2011.02.010

[69] K. Shinohara, Y. Sugii, A. Hibara, M. Tokeshi, T. Kitamori, K. Okamoto, Exp. Fluids **38**, 117–122 (2004). 10.1007/s00348-004-0906-z

[70] A. Hibara, M. Tokeshi, K. Uchiyama, H. Hisamoto, T. Kitamori, Anal. Sci. **17**, 89–93 (2001). 10.2116/analsci.17.89 10.2116/analsci.17.89 11993683

[71] S. Battat, J.T. Ault, S. Khodaparast, H.A. Stone, Soft Matter **15**, 3879–3885 (2019). 10.1039/C9SM00427K 10.1039/c9sm00427k 31021341

