# Supplementary material for: Shaping the gradients driving phoretic micro-swimmers: influence of swimming speed, budget of carbonic acid and environment
Source: Eur Phys J E Soft Matter. 2021 Mar 23;44(3):41. doi: 10.1140/epje/s10189-021-00026-9 (PMC7987694; doi:10.1140/epje/s10189-021-00026-9)
Supplement: Supplementary file 1 — Supplementary material 1 (docx 5733 KB) [file 10189_2021_26_MOESM1_ESM.docx]

Shaping the gradients driving phoretic micro-swimmers: influence of swimming speed, budget of carbonic acid and environment.

Nadir Möller^1,§^, Benno Liebchen^2^, and Thomas Palberg^1^

^1^Institute of Physics, Johannes Gutenberg Universität, Staudinger Weg 7, 55128 Mainz

^2^Institute of Condensed Matter Physics, Technische Universität Darmstadt, Hochschulstr. 8, 64289 Darmstadt

^§^corresponding author, email: namoelle@uni-mainz.de

**Supplementary material**

1) Geometrical considerations for the shape of the measurable pH profile

*Elliptical distortion under flow*


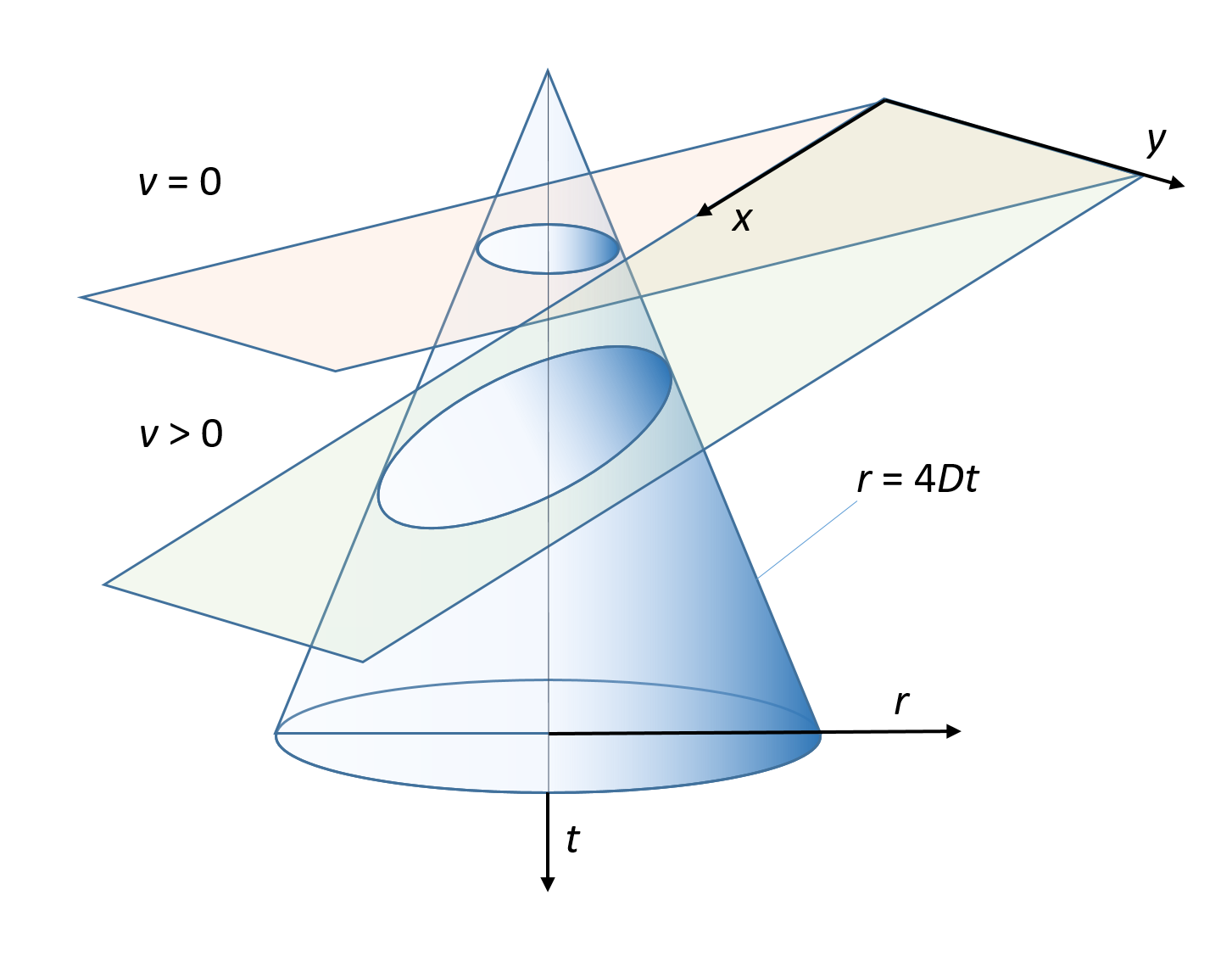


Fig. S1: geometrical considerations concerning the flow profile at source height.

For a radially symmetric purely diffusive transport process, the increase of the radial distance of a concentration contour with time is linear and relates to the mean squared displacement. For a stationary source, its projected shape is circular. This is sketched in Fig. S1. Allowing for a finite and increasing source velocity is equivalent to an increased inclination of the measurement plane. The contour shape then is a conical section, i.e. for not too large velocities it is an ellipse.

2) Exploring the theoretical model

*Profiles and slopes*

To study the influence of the different parameters on source height and averaged pH profiles and slopes, we performed additional calculations. Where not noted otherwise, these are based on a set of typical parameters corresponding to the experimental results obtained for the swimmers on PMMA: *D*_eff_ *= D* = 2000 µm^2^s^-1^; µ ^+^ = µ^+^ = 100 mol L^-1^ s^-1^; µ^−^ = 10^-3^ s^-1^. Another relevant length scale is the typical cargo distances chosen to be *x*_IEX_ – *x*_cargo_ = -50 - -100 µm. We used Eqns. (9) and (10) in Eqn. (12) to calculate the 3D pH distribution, which was then averaged using Eqn. (13) with *H* = 500mm to obtain the profiles. The results are shown in Fig. S2. In each case all input parameters except one are fixed to typical values, The free parameter is varied over an extended large range.

Fig. S2: Profiles along the propulsion direction calculated in a coordinate system such that **e**_x_⎪⎢**v**. a) Profile at source height. Shown are predictions of Eqn. (9) for various velocities as indicated. Note that the velocity only influences the front profile in a significant way. The insert magnifies the region of typical cargo distances, which displays a barely visible dependence on velocity. b) Predictions of height averaged pH profiles for v = 4.1 µm s^-1^ and variable diffusion coefficients as indicated. For large *D*, the shallow minimum (pH = 4.3) is very close to the IEX and the profile shows only a slight asymmetry. With decreasing *D*, the minimum pH drops, the minimum location shifts to the swimmer back and the asymmetry gets very pronounced. For very small *D,* the front profile saturates already very close to the IEX. c) Predictions for v = 4.1 µm s^-1^ and variable loss rates as indicated. The profile changes notably only for loss rates larger than 10^-4^ s^-1^. With increasing µ^−^, the steepness of the profiles is retained, but the asymmetry is markedly reduced. Note, that the location of the minimum is hardly influenced by a change in µ^−^.

A full calculation of the slopes of height averaged pH profiles would afford the numerical derivation of profiles calculated as above. However, here we are here mainly interested in the qualitative behaviour, i.e. the variation of slopes under the influence of the different input parameters. We here used the analytical approximation given in Eqn. (14). This simplification amounts to neglecting the reflection boundary condition at the walls. For selected parameter sets, we checked, that this approach did not alter the data in any qualitative way. Neglect of reflection boundary conditions introduces a scaling factor of slightly larger than 2. This is reasonable, since at *H* = 500µm the main correction in Eqn. (12) stems from the first reflection at the bottom wall. The results are shown in Fig. S3. We here used the same set of input parameters as before. In each case all input parameters except one are fixed to typical values, The free parameter is varied over an extended large range.

Fig. S3: Slopes. a) Slopes of measurable pH profiles predicted by Eqn. (12) as a function of distance to the profile minimum *x*_MIN_ for different velocities as indicated. The vertical dashed lines denote the location of the effective height used in the calculations. Note, that for zero loss rate, the projected back slopes are independent of velocity and decrease as a function of distance. The front slopes increase with increasing velocity and decrease with distance but stay finite out to very large distances. b) The same, but for µ^−^ = 10^-3^ s^-1^. Now, also the back slopes show a small velocity dependence. c) The same for fixed velocity v = 4.1 µm s^-1^ and different µ^−^ as indicated. With increasing µ^−^, the slopes take larger values. This is an important observation for future experiments with systems of finite loss rates by follow-up reactions of the chemicals. d) The same for fixed velocity v = 4.1 µm s^-1^, µ^−^ = 0 and different *D* as indicated. With increasing *D*, the slopes take smaller values. Here, the back slope stays unchanged. This is different for finite µ^−^, where a *D*-dependence obtains, which for µ^−^ = 10^−3^ s^-1^ similar to the one seen for different *v* in Fig. S3b. For larger µ^−^ it gets even more pronounced. For µ^−^ = 1, it is on the same order as the front slope spread µ^−^ = 0 in Fig. S3d

*Slip velocity*

We shortly note some interesting consequences deriving from our exploration. These relate to the phoretic slip velocity at the surface of any charged surface experiencing a finite ground slope, i.e. pH gradient at the level of the object. In gradient direction, the contributions of electro-phoretic (charge driven) and chemi-phoretic (concentration driven) origin are given by [^[[1]](#endnote-1)^, ^[[2]](#endnote-2)^]:

$\mathbf{v}=-\mu\mathbf{E}+\frac{\varepsilon_{0}\varepsilon_{r}}{2\pi\eta}\left(\frac{k_{B}T}{ze}\right)^{2}\ln(1-\xi)\nabla\ln c$ (S1),

where the electro-kinetic mobilities µ_ph, particle_ and µ_ph, substrate_ can be determined from independent experiments [^[[3]](#endnote-3)^]. ε_0_ and ε_r_ are the dielectric permittivity of vacuum and solvent, respectively, η is the solvent viscosity, *k*_B_*T* is the thermal energy, *z* is the valence of the diffusing electrolyte ions, and ξ = tanh(*ze*ζ */ k*_B_*T*), with the surface’s zeta potential ζ. The magnitudes of the electro-phoretic contribution to resulting slip velocites at e.g. the substrate surface or the surface of a potential cargo are, then, *v*_ph, substrate_ = µ_ph, substrate_ *E* and *v*_ph, particle_ = µ_ph, particle_ *E*, respectively. Here *E* is the magnitude of the electric field component in radial direction (appropriately averaged along the substrate, respectively along the projection of the cargo contour). The diffusio-electric field, **E**, results from the different diffusivities of the ions making up the gradient, expressed as diffusivity ratio, β. It ensures global electro-neutrality of the solvent. Specifically, for a 1:1 electrolyte with diffusion coefficients *D*_+_ and *D*˗, like e.g. carbonic acid, it approximately reads [1]:

$\mathbf{E}\approx\frac{k_{B}T}{ze}\frac{D_{+}-D_{-}}{D_{+}+D_{-}}\frac{\nabla c}{c}\equiv\frac{k_{B}T}{ze}\beta\nabla\ln c=\frac{k_{B}T}{ze}\beta\ln(10)\nabla\text{pH}$ (S2),

Presence of the electric field enables swimmer assembly and propulsion [^[[4]](#endnote-4)^]. Using Eqn. (15) with the experimentally determined averages for production and loss rates and for the effective diffusivities, we determined the ground slope at 50 µm distance to the IEX. This distance corresponds to the average distance of the cargo particles to the IEX. The velocity dependence of this quantity is shown in Fig. S4 (left scale). We here used the average values of the diffusion coefficients as experimentally determined for the two substrates investigated. This results in slopes of lager magnitude for the SiO_2_ case. We further observe a weak decrease of the magnitude with velocity. We next used Eqn. (S2) to calculate the diffusio-electric field. This is displayed in Fig. S4.

Fig. S4 Ground pH gradients of swimmers at *x* = *x*_IEX_ – *x*_cargo_ = -50 µm for different speeds using Eqn. (15). Calculations were performed using experimental data for the diffusion coefficient for each surface substrate (*D*_PMMA_ = (2002±132) µm^2^s^-1^ and *D*_SiO2_ = (1585±142) µm^2^s^-1^) and the average loss rate µ^-^ = 10^-3^ s^-1^. The right-hand axis denotes the corresponding diffusio-electric field given by Eqn. (S2) (Note the negative sign of both quantities emerging from the negative x-values considered and **e**_x_⎪⎢**v**).

The field magnitudes compare well to those in previous work [^[[5]](#endnote-5)^]. Given this field strength and an electro-phoretic mobility of the cargo of µ_ep_ = 2.1 µm s^-1^ / V cm^-1^ we have a solvent speed directed along the cargo surface of 9-10 µm/s. For each assembled cargo such a “blow” impinges locally on the spherical IEX sail, with an efficiency <1. Still, the individual contributions add up near linearly. This resulting in a stepwise swimmer increase of swimmer velocities up to values of a few microns per second (see [^[[6]](#endnote-6)^] and Fig. S4, below). The first step is somewhat larger. This may be expected from the slight decrease of the field with increasing velocity, but moreover from the geometrical observation, that neigbouring cargo will “share” the blow occurring at their adjacent side.

Further, even for large distances, the absolute values of both ground slopes remain finite, and the relative changes for increasing loss rate and/or velocity get larger. As simulations for a stationary source have shown, the electro-osmotic slip, then, gives rise to long-ranged solvent flows. For cylindrical cells of finite height a stationary convection pattern forms featuring two toroidal cells, counter-rotating and stacked on top of each other [^[[7]](#endnote-7)^]. Moreover, it also opens the possibility for mutual swimmer-swimmer interactions via diffusio-phoretic effects. These have been seen in different experiments and may occur even where the gradients are too small to be detected in fluorescence measurements or in height averaged photometry [^[[8]](#endnote-8)^]. Consequently, the interaction strength should depend both system chemistry and swimmer velocity. Further, the interaction strength directly relates to the diffusivity ratio and should switch sign from effectively attractive to effectively repulsive, e.g. when going from gradients in HCl to gradients of NaOH.

3) Selecting swimmers suitable for quantitative analysis

In Fig. S5, we display individual measurements of the cargo number dependent swimmer velocity for different swimmer types on different substrates. Since we did not average over a larger number of identical swimmers, we observe a large statistical scatter. Still the displayed exemplary data yield characteristically different curves for the three chosen combinations. In all cases, the swimming velocity increases systematically and roughly linearly with the number of assembled cargo particles. At still larger *N*, the velocity reaches a constant value.


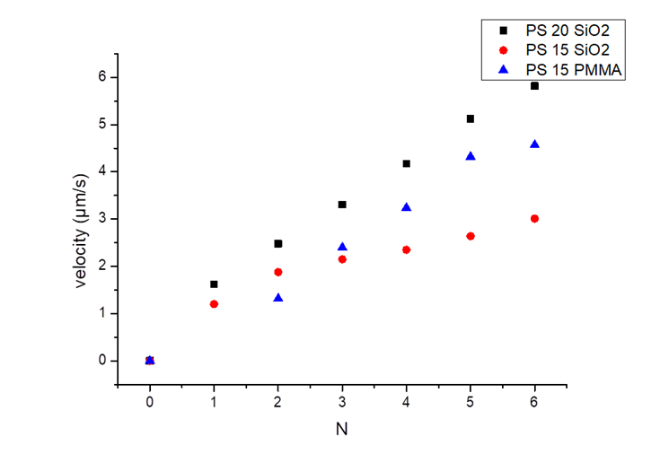


Fig. S5: Cargo number dependent swimmer speeds determined from tracking analysis for selected swimmers. As colour coded in the key, data are for different combinations of IEX45 and cargo of different sizes (PS15, PS20) and with different substrates.

Not all swimmers qualify equally for further quantitative analysis. To identify those suitable, we here addressed the effects of neighbouring swimmers and cargo uptake on pH maps, pH field shape and collinear pH profiles

**

Fig. S6: examplary pH maps of swimmers rejected for further quantitative analysis. a) overlap of pH fields with those of neighbouring swimmers; b) non straight trajectories due to cargo-upload.

In several cases we found two swimmers in vicinity to each other and even in the same image. This has a great influence on the pH maps and on profiling. Note, for example in Fig. S6a, the distortion of the elliptic region of 5.0 < pH < 5.2 and the presence of an additional region for these values at the lower right (as well as a small pH decrease at the upper right image boundary). Such patches result from the simultaneous presence of additional sources. Care was to be taken to select only those pH map videos for quantitative analysis, where the swimmer crosses a homogeneous and pristine, undisturbed environment. Even so, other artefacts may occur, most often due to cargo pick-up. In such cases, the swimmer typically changes both speed and direction. Minor direction changes may occur upon cargo rearrangement. The effects of the former type of event can be seen in Fig. S6b. Here, the swimmer had taken up a second cargo bead in the upper left corner some 300s ago. This was correlated to a turn in its trajectory (white line) and an increase of ground speed. Note, how the pH contours of large valued pH intervals with pH ≥ 5.0 are distorted upward, while contours of low valued pH intervals evolved undisturbed. We here also show fitted ellipses for the outer contours of regions showing values of pH ≤ 4.8 (black); pH ≤ 5.0 (red) and ≤ 5.2 (blue). While the former two are aligned with the current propulsion direction, the latter is turned clockwise by a considerable angle. The contours of larger valued pH regions aren’t described by ellipses any more.

Fig. S7a compares the profiles of straight (blue) and non-straight swimming swimmers (red) on the pH-profile. The straight swimmer shows a linear increase of the pH both to the fromnt and to the back. The profile of the non-straight swimmer (which is the one from Fig. S6b) shows a distinct kinks in the profile corresponding to the cargo pick-up and the change in direction. This is also sketched again in the inset. Maps and profiles with such signatures were excluded from further evaluation.

In some rare cases, swimmers picked up a further cargo bead without significant change of propulsion direction. This allowed to study the effect of acceleration upon cargo on the pH field shape, as derived from ellipses fitted to the pH contours, which here stayed well aligned. In Fig. 7b, we show evolution of the ratio of major to minor axis after picking a second cargo bead at a covered distance of about 430µm. Exemplary error bars give the standard error of the fits at a confidence level of 0.95. Before uptake, the shape appears to stay stationary within fitting accuracy. Upon uptake, the pH contour closest to the swimmer starts transforming immediately, but there is some time lag for the outer contours. The important finding, here, is that whenever the cargo number changes, a stationary state evolves again in short times and over relatively short travel distances (here, some 500µm). We therefore restrict systematic analysis to straight trajectories of constant cargo number and of sufficiently long duration.

Fig. S7: Effects of cargo pick-up. a) comparison of pH profiles for straight and non-straight swimming. b) Evolution of the ratio of major to minor axis of ellipses fitted to the outer contours of the pH values indicated as a function of covered distance for the rare event of cargo uptake *without* significant directional change. The vertical dotted line at 430 µm denotes the location of the uptake.

4) Additional data from pH-field shape analysis

In Fig.S8a and b we display the results of shape characterization in terms of relevant geometric quantities in dependence on swimmer velocity.

Fig. S8: a) Averaged ratio of major to minor axis of ellipses, fitted to the outer contours of the pH values indicated, for four different ground speeds. Error bars correspond to the standard deviation of fit results for each speed. This ratio increases roughly linearly with speed in this velocity range. b) Average distance from the centre of mass of the IEX beads to the front focal points of ellipses fitted to the outer contours of the pH values indicated for the same ground speeds as in a). The inset shows the geometry underlying this calculation. We find that the centre of the ellipses (*M*) stays well behind the IEX as expected from Fig. S1. Further, the front focus of the ellipses is slightly displaced from the position of the IEX bead. At low speed, this difference is almost negligible for the innermost contour, but clearly seen for the outer ones. In all cases, the difference increases with increasing speed.

In Fig. S9a, we show the temporal evolution of the ratio of major to minor axis for such a swimmer. Error bars give the standard error of the fits at a confidence level of 0.95. There is some systematic fluctuation, in particular for the innermost contour, which was traced back to minor cargo rearrangements with respect to the propulsion direction. The outer contours are less affected by this effect. There, statistical scatter dominates. Overall, Fig. 10a demonstrates a remarkable shape stability of the pH profile in time, i.e. it confirms the theoretically expected existence of a stationary state.

Fig. S9: pH field characterization with color-coding as in Fig. 7b and repeated in the key. a) Evolution of the ratio of semimajor to semiminor axis of ellipses fitted to iso-pH contours as a function of covered distance. b) Averaged eccentricity of ellipses fitted to iso-pH contours as a function of velocity.

In line with previous studies [4], we observed that for each combination of IEX bead size, cargo size and cargo number, a characteristic speed was obtained. To improve statistics, we monitor up to five swimmers for each speed, perform iso-pH contour fits for each after several different covered distances. In Fig. S9b we display the averaged eccentricity, *e*, as a function of ground speed, where *e* is the ratio of the distance between the centre of the ellipse and each focus to the length of the semimajor axis *a* of the ellipses. Error bars correspond to the standard deviation of the obtained averages. Even small velocities distort the elliptical iso-pH contours to relatively large eccentricities *e*, Moreover, *e* increases with increasing pH, i.e. at low speed, outer contours are significantly more affected by head winds than inner ones, while at large speed this differences decrease.

5) Additional data from pH profile analysis

In Fig. S8, we display exemplary profiles for two single cargo swimmers on SiO_2_ exhibiting different ground speeds (here due to different IEX/cargo size ratios). The slower swimmer (black) shows the steeper increase of pH at the back (left) and a lower one at the front (right). The faster swimmer (red) shows an initially steep increase of pH at the front but then the increase slows due to the onset of saturation effect. Also shown are straight lines as guides to the eye. A linear increase of pH is seen over an extended distance interval until saturation effects interfere.

Fig. S10: Comparison of exemplary profiles for two swimmers on SiO_2_. Insets depict top view sketches of the swimmers. Straight lines are guides to the eye.

We have measured the collinear, height-averaged pH-profile of swimmers in different scenarios. Fig. S10 and S11 give further examples with the corresponding fits of our model using Eqn. (10) + (12). Profiles were recorded for swimmers propelling with different velocities on glass and on PMMA substrates under otherwise identical boundary conditions. In all cases the model describes the data very well, and the characteristic parameters (production and loss rates, effective diffusion coefficient) can be extracted with good precision. Interestingly, the back slope is also well described in case of cargo pickup, but only over shortened distance [Fig. S11b). See also the discussion in section 3.

Fig. S11: Comparison of exemplary profiles for swimmers in different scenarios. The fits correspond to height-averaged curves using Eqn. (10) + (12). a.) pH profile of a swimmer on a glass substrate, *D* = (1602±24) µm^2^s^-1^. b.) Swimmer moving on a PMMA substrate, *D* = (2012±98) µm^2^s^-1^. Note the mismatch between fit and data for x < -800µm due to cargo pickup c.) Same as a) but at with a higher velocity. *D* = (1508±51) µm^2^s^-1^. d.) pH curve of a swimmer on a glass substrate that move inside a more restrictive cell of height H = 250 µm, *D* = (1448±142) µm^2^s^-1^.

To highlight the performance and importance of the confinement originating from cell height on the, we also recorded the pH profile of a swimmer on a glass substrate with a cell of height of H = 250 µm. Again, the profile is fitted well and the extracted parameters agree well to those obtained for other cell heights.

In Fig. S12, we demonstrate the failure of the using Eqns. (5) and (6) for pumps in a quiescent solvent in Eqn. (12) for projection and confinement correction in profile fitting for a pump working on SiO_2_.

Fig. S12: single free parameter fitting of pump profiles using Eqns. (5) and (6) for pumps in a quiescent solvent in Eqn. (12). For PMMA (red diamonds), we fixed the inputs to the averages obtained from the swimmer experiments on this substrate: *D*_PMMA_ = 2002 µm^2^s^-1^, and µ^-^ =1.2×10^-3^ s^-1^. We further set *t* = 200 s (corresponding to the time at which the pH map was recorded), *c*_∞_ = 3.24 mol L^-1^ and *v* = 0 µms^-1^. This fitting works well. For the free parameter we obtain µ^+^ = (153±12) mol L^-1^ s^-1^ agreeing with the previously determined average value with the experimental scatter (arising from slightly differing IEX sizes). By contrast, a fit to the SiO_2_ data using D_SiO2_ = 1585 µm^2^s^-1^ fails to describe the data. This is due to the neglect of solvent advection, which for this substrate is considerably more pronounced. However, as seen in the main part of the manuscript, the special case of inhomogeneous but radially symmetric solvent advection can be incorporated and parameterized using an effective velocity equivalent to homogeneous net solvent inflow.

1. J. L. Anderson, Ann. Rev. Fluid Mech. 21, 61-99 (1989). [↑](#endnote-ref-1)
2. J. L. Anderson and D. C. Prieve, Separation & Purification Reviews **13**, 67-103 (1984). [↑](#endnote-ref-2)
3. D. Botin, J. Wenzel, R. Niu, T. Palberg, *Soft Matter* **14**, 8191-8204 (2018). [↑](#endnote-ref-3)
4. B. Liebchen, R. Niu, T. Palberg, H. Löwen, Phys. Rev. E **98**, 052610 (2018). [↑](#endnote-ref-4)
5. A. A. Farniya, M. J. Esplandiu, D. Reguera, and A. Bachtold, *Phys. Rev. Lett.* **111**, 168301 (2013). [↑](#endnote-ref-5)
6. R. Niu, D. Botin, A. Reinmüller, T. Palberg, *Langmuir* **33**, 3450-3457 (2017). [↑](#endnote-ref-6)
7. R. Niu, P. Kreissl, A. T. Brown, G. Rempfer, D. Botin, C. Holm, T. Palberg, J. de Graaf, *Soft Matter* **13**, 1505-1518 (2017). [↑](#endnote-ref-7)
8. R. Niu, T. Palberg *Soft Matter* **14**, 7554-7568 (2018). [↑](#endnote-ref-8)
